# Rapid bacterioplankton transcription cascades regulate organic matter utilization during phytoplankton bloom progression in a coastal upwelling system

**DOI:** 10.1038/s41396-022-01273-0

**Published:** 2022-07-08

**Authors:** Benjamin Pontiller, Sandra Martínez-García, Vanessa Joglar, Dennis Amnebrink, Clara Pérez-Martínez, José M. González, Daniel Lundin, Emilio Fernández, Eva Teira, Jarone Pinhassi

**Affiliations:** 1 Centre for Ecology and Evolution in Microbial model Systems – EEMiS, Linnaeus University, 39182 Kalmar, Sweden; 2 GEOMAR Helmholtz Centre for Ocean Research Kiel, 24105 Kiel, Germany; 3 Centro de Investigación Mariña da Universidade de Vigo (CIM-UVigo), Departamento de Ecoloxía e Bioloxía Animal, Universidade de Vigo, Campus Lagoas-Marcosende, 36310 Vigo, Spain; 4 Department of Microbiology, University of La Laguna, 38200 La Laguna, Spain

**Keywords:** Transcriptomics, Water microbiology, Microbiome, Microbial ecology, Biogeochemistry

## Abstract

Coastal upwelling zones are hotspots of oceanic productivity, driven by phytoplankton photosynthesis. Bacteria, in turn, grow on and are the principal remineralizers of dissolved organic matter (DOM) produced in aquatic ecosystems. However, the molecular processes that key bacterial taxa employ to regulate the turnover of phytoplankton-derived DOM are not well understood. We therefore carried out comparative time-series metatranscriptome analyses of bacterioplankton in the Northwest Iberian upwelling system, using parallel sampling of seawater and mesocosms with in situ-like conditions. The mesocosm experiment uncovered a taxon-specific progression of transcriptional responses from bloom development (characterized by a diverse set of taxa in the orders *Cellvibrionales*, *Rhodobacterales*, and *Pelagibacterales*), over early decay (mainly taxa in the *Alteromonadales* and *Flavobacteriales*), to senescence phases (*Flavobacteriales* and *Saprospirales* taxa). Pronounced order-specific differences in the transcription of glycoside hydrolases, peptidases, and transporters were found, supporting that functional resource partitioning is dynamically structured by temporal changes in available DOM. In addition, comparative analysis of mesocosm and field samples revealed a high degree of metabolic plasticity in the degradation and uptake of carbohydrates and nitrogen-rich compounds, suggesting these gene systems critically contribute to modulating the stoichiometry of the labile DOM pool. Our findings suggest that cascades of transcriptional responses in gene systems for the utilization of organic matter and nutrients largely shape the fate of organic matter on the time scales typical of upwelling-driven phytoplankton blooms.

## Introduction

Wind-induced upwelling of nutrient-rich subsurface water triggers pronounced phytoplankton blooms along eastern boundary coastal zones. Despite their small surface area (~7% of global ocean), coastal seas contribute 14-30% of the global oceanic primary production [1]. Phytoplankton produce a diverse mixture of organic compounds by photosynthesis [2–4], which is rapidly processed by prokaryotes into biomass and CO_2_ [5]. Typically, substrate availability and interactions among microorganisms determine spatiotemporal differences in microbial community composition and function [6–11]. However, compared to the temporal dynamics in bacterial community composition [12, 13], little is known about the microbially mediated processes involved in the degradation and uptake of phytoplankton-derived organic matter on the time scales of a few days to weeks that characterize upwelling blooms.

Although bacterioplankton communities are composed of thousands of bacterial populations, typically only a few become dominant during phytoplankton blooms [6]. Hence, it has been postulated that the utilization of phytoplankton-derived dissolved organic matter (DOM) is partitioned among specialized bacteria that thrive under rapidly changing environmental conditions [6]. This is underscored by both field studies of naturally occurring phytoplankton blooms [11, 14–17] and enrichment experiments [18–21]. For example, well-known phytoplankton-associated bacterial taxa such as the *Flavobacteriaceae* and *Alteromonadaceae* are efficient degraders of algal polysaccharides, proteins, and glycoproteins [6, 11, 13], and the Roseobacter clade (*Rhodobacteraceae*) are exceptionally competitive utilizers of low-molecular-weight DOM compounds like dimethylsulfoniopropionate (DMSP), polyamines, and taurine [6, 22]. Collectively, these studies show that DOM compositional characteristics are important for structuring bacterioplankton community composition, potentially driving bacterial succession over longer time scales.

Several studies that investigated bacterial responses to phytoplankton-derived organic matter [18, 23–26], concentrated seawater DOM [27, 28] and DOM model compounds [4, 29, 30], found pronounced functional resource partitioning among distinct bacterial clades. Metabolic functions that are typically detected in bloom-associated bacteria involve the degradation of polymers (e.g., carbohydrate-active enzymes that hydrolyze glycosidic bonds found in polysaccharides or proteolytic enzymes that hydrolyze peptide bonds), and the transport of hydrolysis products, besides features like surface adhesion, bacterial signaling, biofilm formation, and motility [11, 24]. While there is ample knowledge of the molecular mechanisms driving DOM utilization by bacteria in the open ocean, corresponding knowledge for coastal seas is limited.

Given the rapidly shifting phytoplankton bloom dynamics in upwelling areas, we carried out a shipboard mesocosm experiment over seven days to investigate bacterial growth and transcription responses associated with different bloom phases (i.e., bloom development, through early decay to senescence phases). In parallel with the experiment, we sampled the field station from where the mesocosm water was collected, to contrast the responses measured in the experiment with dynamics taking place in the natural environment. We hereby hypothesize that the detection of transcriptional differences between key taxa across bloom phases in a replicated experimental setting would have the potential to inform on the ecology of these taxa in their natural environment.

## Materials and methods

### Study site, sampling, and experimental setup

Seawater was collected during the ENVISION-III cruise [31], at shelf station 3 (Stn 3; 42° 7’ 42.3984” N, 8° 55’ 44.9724” W) (Fig. S1). Detailed methods on the experimental setup are given in Supplementary Material. Briefly, for initiating the mesocosm experiment on 5 August 2016 (day 0), each of three mesocosms received a mix of nutrient-rich water from 20 m depth (152 L; derived from a recent upwelling) and water from 5 m (38 L; with a phytoplankton bloom under development) (Fig. S2). All water was filtered through a 200 µm mesh. Based on previous experience [32], we expected this would induce pronounced and replicated phytoplankton blooms in the mesocosms, of comparable magnitude to the blooms occurring in natural surface waters. This experimental design allowed us to determine the transcriptional responses of bacteria to different bloom phases, by avoiding advective processes that would disrupt the temporal connectivity between samples sequentially collected at sea. Mesocosms were incubated onboard and to maintain in situ temperature (~15 °C) the mesocosms were placed in a tank with flow-through seawater from ~2 m depth. Subsamples of ~5 L were collected daily for total prokaryotic cell counts and ^3^H-leucine incorporation rates, chlorophyll *a*, and NH_4_^+^, NO_2_^−^, NO_3_^−^, PO_4_^3−^, and SiO_4_^4−^ concentrations, and on days 0, 1, 3, 5, and 7 for DNA and RNA extractions. In parallel, corresponding samples were collected from 5 m depth at Stn 3.

### Chlorophyll *a* and nutrients

Size-fractionated chlorophyll *a* (Chl *a*) concentrations were determined by sequentially filtering 300 mL seawater samples through 3.0- and 0.2-μm-pore-size polycarbonate filters [31]. Chl *a* was extracted with 90% acetone at 4 °C overnight in the dark. Fluorescence was determined using the non-acidification technique [33], with a TD-700 fluorometer (Turner Designs) calibrated with a pure Chl *a* solution [34]. Samples for inorganic nutrient analysis (NH_4_^+^, NO_2_^−^, NO_3_^−^, PO_4_^3−^, and SiO_4_^4−^) were collected in 50 mL polyethylene bottles and stored at –20 °C until analysis by standard colorimetric methods [35]. Dissolved organic carbon (DOC) concentrations were measured with a Shimadzu TOC-V analyzer according to [36] (Supplementary Material).

### Prokaryote abundance and heterotrophic production

Samples (1.8 mL) for prokaryote abundance were preserved with a mix of 1% and 0.05% (final concentrations) of paraformaldehyde and glutaraldehyde, respectively, and frozen at −80 °C until analysis. Samples were stained with 2.5 mM SybrGreen DNA fluorochrome and enumerated with a FACSCalibur flow cytometer (Becton Dickinson) [37]. Bacterial heterotrophic production was estimated through the ^3^H-leucine incorporation method according to [38]. Samples (1 mL in triplicates) were amended with 40 nM radioactive leucine (final concentration) and dark-incubated at in situ temperature for 1 h. Production was calculated using a conversion factor of 3.1 kg C mol Leu^−1^ [38] (Supplementary Material).

### Microbial community composition analysis

Prokaryotic and eukaryotic community composition were determined from ~2 L water samples, which were sequentially filtered through 3-μm-pore-size Nuclepore polycarbonate filters (Whatman) and 0.22-μm-pore-size Sterivex filters (EMD Millipore) and analyzed as described in [31] and Supplementary Material. In brief, for prokaryotes, the 16S rRNA gene V4-V5 region was amplified using the universal primers “515 F” and “926 R” [39] from the <3.0-µm and >0.2-µm size fraction. Eukaryotic 18S rRNA genes were amplified using the primers TAReuk454FWD1 and TAReukREV3 [40] from both size fractions. Amplicons were sequenced on a MiSeq platform (Illumina, Inc.) to obtain 2 × 300 bp paired-end reads. Raw reads were processed using the Ampliseq (v2.2.0) pipeline [41] and SILVA reference database (v138.1) [42] for taxonomic assignments of 16S rRNA gene amplicon sequence variants (ASVs). The databases PR2 together with the marine protist database from the BioMArKs project were used to infer taxonomy of 18S rRNA gene ASVs.

### Metatranscriptomics analysis

For metatranscriptomics, ~3.5 L water samples from each of the triplicate mesocosms and the field samples were sequentially filtered through 3-μm-pore-size polycarbonate filters (Whatman) and collected on Sterivex filters (GP 0.22-μm-pore-size), preserved in 2 mL RNAlater (Qiagen, Hilden, Germany), and immediately flash-frozen in liquid nitrogen. RNA was extracted using RNeasy (Qiagen), treated to remove DNA and rRNA, and linearly amplified with minor modifications [29, 43]. Sequencing was done at the Swedish National Genome Infrastructure, on a HiSeq 2500 platform (Illumina, Inc.) in rapid mode to obtain 2 × 125 bp paired-end reads. Sequencing summary statistics are in Table S1.

Details on metatranscriptomics analyses are in Supplementary Material. In brief, Illumina adapter sequences were removed with Cutadapt [44] and reads were trimmed with Sickle using default settings [45]. Reads aligning to an in-house database of stable RNA sequences were removed with ERNE [46] and quality reads de-novo assembled with MEGAHIT [47] separately for mesocosm and field samples. Open reading frames (ORFs) were determined with Prodigal [48] in single mode. The ORFs were clustered at a 99% level with VSEARCH [49] and aligned to the NCBI Refseq protein database (release date: December 20, 2018) with DIAMOND [50]. Taxonomic annotations were assigned using MEGAN [51]. For details on annotations and analyses of transcribed DOM active genes and phylogenetic marker genes see Supplementary Material. In brief, glycoside hydrolases (GHs), peptidases (PEPs), transporters (TPs), and sulfatases (STs) were detected and classified with HMMER3 using HMM profiles specific for each in the PFAM, MEROPS, Transporter Classification, and SulfAtlas databases, respectively. GHs were additionally classified with run-dbcan (v2.0.11) against the dbCAN2 database (release date: July 31, 2018) [52]). To further identify transcriptionally active taxa, we carried out phylogenetic analyses on two expressed marker genes broadly distributed in bacteria: the genes coding for ribosomal protein L12 (the most highly expressed ribosomal protein gene in our data set) and for RecA (necessary for maintaining DNA integrity).

### Statistics, normalizations and visualization

Detailed description of statistics and normalizations are provided in the Supplementary Material. In brief, for principal component analysis (PCA), raw counts were transformed into centered log ratios (clr) using *CoDaSeq* [53] (v0.99.6) and Euclidean distances computed with the function *dist* (vegan v2.5–7). PCAs were performed using the function *prcomp* (*stats* v4.1.0) in R v4.1.0 [54]. For order-specific PCA analyses, clrs were calculated for each order separately. Redundancy analysis (RDA) was performed on the same input data as described above (clr). Environmental variables used in the RDA were selected based on pairwise Pearson correlations coefficients <0.9 and variance inflation factors <10. To detect differences in gene transcription between bacterial orders, we normalized individual gene transcript counts to the total transcription within each order, attempting to favor changes in bacterial transcription over changes in abundance. The same principle was applied to analyses at the genus level, whereby genus level information on transcription of the studied gene systems was obtained by grouping order-normalized transcript counts at the respective taxonomic genus levels and functional GH family or PFAM levels (Supplementary Material).

For visualization in Ternary plots, we grouped order-normalized transcript counts at the genus and GH family or PFAM level into development phase (DP - mean of day 0 and day 1; *n* = 4), early decay (ED - mean of day 3; *n* = 3), and senescence phase (SP - mean of day 5 and 7; *n* = 6), and standardized the counts to equal row sums (Supplementary Material). To obtain additional insight into the transcriptional dynamics of the coastal upwelling system, we compared the expression of these genes at GH family and PFAM levels between the parallel mesocosm and field samples through linear regressions based on log2-transformed order-normalized transcripts (Supplementary Material).

## Results

### Microbial dynamics during an upwelling-driven phytoplankton bloom

**Fig. 1:**
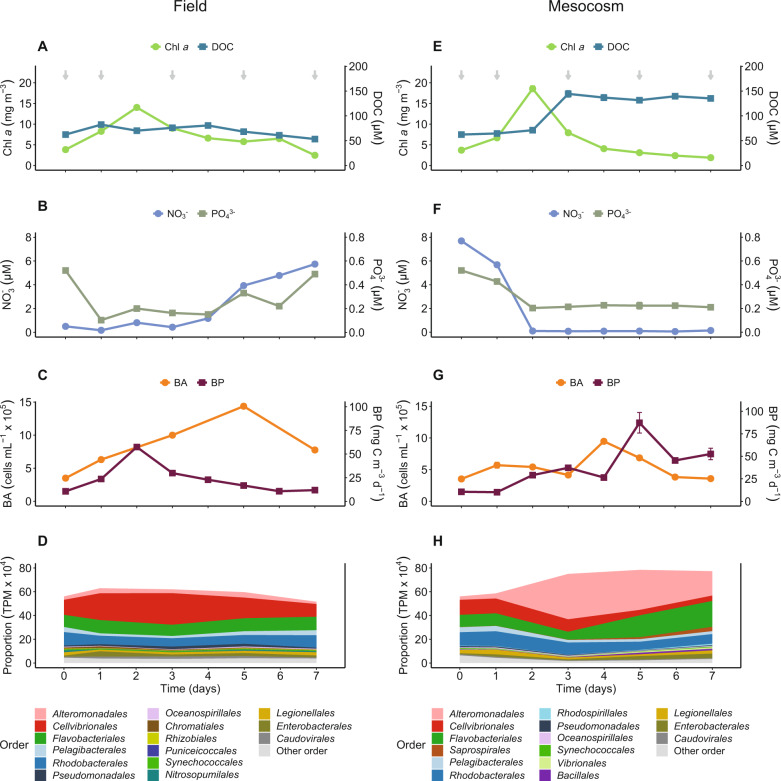
Comparison of the temporal dynamics of biotic and abiotic parameters between field and mesocosm samples. **A**, **E** Chlorophyll *a* (Chl *a*) and dissolved organic carbon (DOC) concentrations, (**B**, **F**) Nitrate (NO_3_^−^) and phosphate (PO_4_^3−^) concentrations, (**C**, **G**) Bacterial abundance (BA) and bacterial production (BP), and (**D**, **H**) Transcription of the 15 most active prokaryotic taxa. Arrows indicate the time points when metatranscriptome samples were taken. Error bars denote standard deviation of triplicate mesocosms, except for day 0, which were taken from pooled water used for filling the tanks (*n* = 1).

Sampling in the NW Iberian Peninsula coastal upwelling system captured a phytoplankton bloom during its development and subsequent decaying phases (Fig. S1). Chl *a* increased from 3.8 mg m^−3^ to a peak of 14 mg m^−3^ on day 2, progressively decreasing to 2.5 mg m^−3^ on day 7 (Fig. 1A). The 18S rRNA gene analysis showed that dinoflagellates (mostly *Dinophyceae*) were dominant components of the eukaryotic community along with ciliates (*Ciliophora*) during bloom development. From day 5 onwards, the relative abundance of diatoms (e.g., *Chaetoceros* and *Thalassiosira*) increased (Fig. S3A, [31]). DOC concentrations ranged from 82 µM C (day 2) to 53 µM C (day 7) (Fig. 1A). The continuous increase in inorganic nutrient concentrations measured from day 4 onwards at station 3 indicated an upwelling pulse (Fig. 1B, Fig. S2). Bacterial production was highest on day 2, coinciding with the phytoplankton peak, and bacterial abundance increased until day 5 (Fig. 1C).

The 16S rRNA gene analysis showed that the bacterial community was largely dominated by e.g., the *Flavobacteriaceae* genera *Aurantivirga* and *Polaribacter* making up ~11% of the community along with *Flavicella* and *Formosa* (up to 6%) and the *Rhodobacteraceae* genera *Planktomarina* and *Yoonia*-*Loktanella* accounting for 8.6% and 6.1%, respectively. *Gammaproteobacteria* such as the genus *Glaciecola* and the clades SAR92 and OM60/NOR5 showed relative abundances ≤2% (Fig. S3B).

The prokaryotic community transcription was dominated by *Cellvibrionales*, which nearly doubled to ~30% of total transcripts on day 3 (Fig. 1D), primarily due to the families *Porticoccaceae* and *Halieaceae* (identified through phylogenetic analyses of the genes encoding ribosomal protein L12 and RecA; Figs. S4, S5). The other taxa remained fairly stable, with *Flavobacteriales* (mainly the *Flavobacteriaceae* genera *Polaribacter* and *Tenacibaculum*; Figs. S6, S7) and *Rhodobacterales* (genus *Planktomarina*; Figs. S8, S9) each contributing ~10% of total transcription. *Pelagibacterales* (genus *Pelagibacter*; Figs. S8, S9) and *Alteromonadales* (genera *Alteromonas* and *Glaciecola*; Figs. S4, S5) accounted for ~4% each (Fig. 1D).

### Microbial dynamics during an experimental mesocosm bloom

As in the field, Chl *a* peaked on day 2 (~18.6 mg m^−3^) and decreased to 1.5 mg m^−3^ on day 7 (Fig. 1E). 18S rRNA gene analysis showed a mixed phytoplankton community, primarily composed of dinoflagellates (mostly *Dinophyceae*) and diatoms (e.g., *Chaetoceros* and *Thalassiosira*) (Fig. S3A). *Chlorophyta* peaked on day 3 and marine alveolates (MALV), and marine stramenopiles (MAST) increased on day 7 (Fig. S3A). The transition from bloom development to early decay was associated with pronounced changes in nutrient concentrations (Figs. 1E, F,  S2). Upon the Chl *a* decrease, dissolved organic carbon (DOC) doubled from day 2 to 3, reaching ~145 µM C (Fig. 1E), whereas dissolved inorganic nutrients sharply decreased (Figs. 1F,  S2). Still, bacterial production reached maximum rates later than in the field, peaking on day 5 - one day after the peak in bacterial abundance (Fig. 1G).

The 16S rRNA gene analysis showed that the genera *Alteromonas* (*Alteromonadaceae*) and *Pseudoalteromonas* (*Pseudoalteromonadaceae*) increased substantially on days 3 and 5 (up to ~10% of community) along with *Rhodobacteraceae* genera like *Planktomarina* and *Yoonia*-*Loktanella* (up to ~14%) (Fig. S3B). During bloom senescence, the *Flavobacteriales* genus *Polaribacter* (*Flavobacteriaceae*) became dominant (up to ~40% on day 7).

The prokaryotic transcriptional responses in the mesocosms were comparable to those in the field during the phytoplankton bloom development phase (days 0 and 1; Fig. 1D, H, S10); dominated by *Cellvibrionales* (families *Porticoccaceae* and *Halieaceae*; Figs. S4, S5), *Rhodobacterales* (family *Rhodobacteraceae* and genus *Planktomarina*; Figs. S8, S9), and *Flavobacteriales* (*Flavobacteriaceae* genera *Polaribacter* and *Tenacibaculum*; Figs. S6, S7). The transition from bloom development to early decay, characterized by the rapid decrease in Chl *a* along with inorganic nutrients and a concomitant increase in DOC until day 3 (Fig. 1E), substantially induced *Alteromonadales* transcription (from 3% to 38% of total transcripts; Fig. 1H), mainly the genera *Alteromonas* and *Glaciecola* (*Alteromonadaceae*) and *Pseudoalteromonas* (*Pseudoalteromonadaceae*) (Figs. S4, S5). At this time, the transcription of both *Flavobacteriales* and *Rhodobacterales* remained fairly stable. Thereafter, *Alteromonadales* transcription decreased to 20% on day 7, whereas *Flavobacteriales* transcription increased to 22% of transcripts. Simultaneously, a few orders with initially low transcription (e.g., *Saprospirales* and *Vibrionales*) increased toward day 7 (Fig. 1H). Bacterial richness and Shannon diversity based on the transcriptional data on the phylogenetic marker genes for L12 and RecA in the mesocosms were highest during bloom development and decreased during bloom senescence. In the field, dynamics in diversity were less pronounced (Fig. S11).

**Fig. 2:**
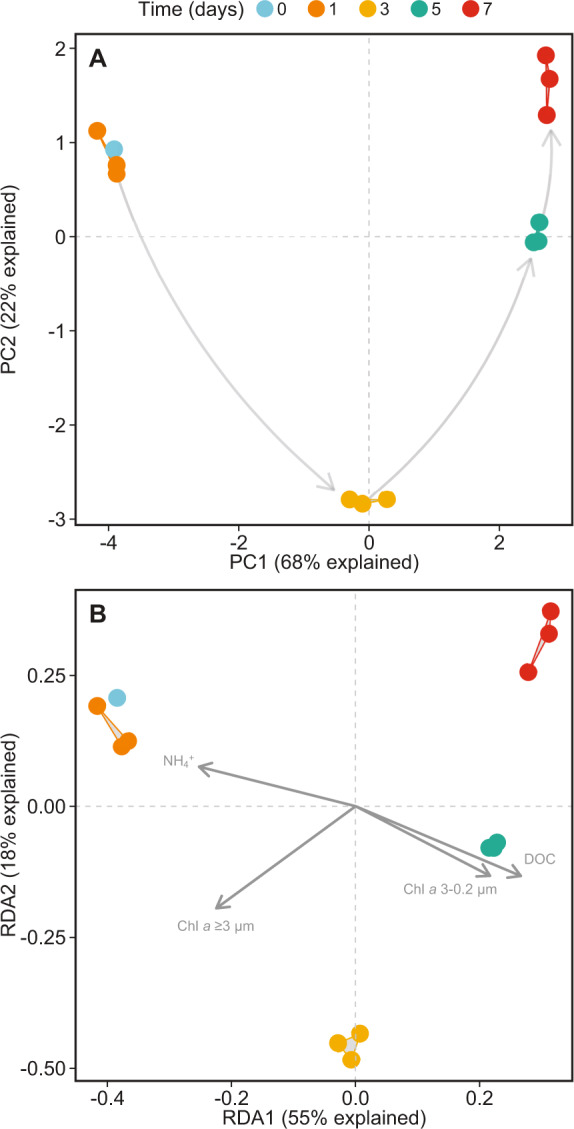
Analysis of changes in prokaryotic transcription during the mesocosm experiment. **A** Principal component analysis (PCA) of clr-transformed transcript counts and pairwise Euclidean distances of open reading frames (ORFs) with at least 5 counts per million (CPM) in at least 3 samples (50533 ORFs accounting for ~68% of total transcripts - TPM). Values were scaled to unit variance. **B** Redundancy analysis (RDA) based on the same data and preprocessing steps. Environmental variables were log-transformed, centered, and scaled to unit variance prior to RDA analysis.

A principal component analysis (PCA) performed on the metatranscriptomic data further emphasized shifts from the bloom development phase (day 0 and 1) to the early decay phase (day 3) and to the senescence phase (day 5 and 7) (Fig. 2A; PC1 explained 68% of the temporal transcriptional variation). The temporal shift in prokaryotic transcription was significant (PERMANOVA, *R*^2^ = 0.79, *p* < 0.001). A redundancy analysis (RDA) showed that Chl *a* (0.2 and 3.0 µm size fractions), DOC, and NH_4_^+^, explained ~55% of the variation on RDA1 and ~19% on RDA2, and collectively accounted for ~68% of the variation in community transcription (Monte Carlo permutation test, *p* = 0.001). Variance partitioning analysis further showed that Chl *a* (3.0 µm fraction) explained ~27% and DOC ~11% of variation in transcription (Chl *a* 3.0–0.2 µm and NH_4_^+^ were not significant) (Figs. 2B,  S12), emphasizing the coupling between phytoplankton bloom development and bacterial gene expression.

### Dynamics in DOM utilization gene transcription in the mesocosm bloom phases

We performed PCAs on order-normalized expression that distinguishes the contribution of transcriptional regulation from changes due to growth (Fig. S13). These analyses showed that *Alteromonadales* expression shifted strongly between day 1 and 3 (Fig. S13A), as did *Flavobacteriales* and *Saprospirales* to a somewhat lesser degree (Fig. S13B, C), largely mirroring the changes in community transcription (Fig. 1H). In contrast, the expression of *Rhodobacterales*, *Cellvibrionales*, and *Pelagibacterales* remained relatively stable until day 5 (Fig. S13D–F), indicating little immediate responsiveness to the DOC increase on day 3. This indicated a divergence between orders in sensing and utilizing changes in the organic matter pool during phytoplankton bloom progression.

**Fig. 3:**
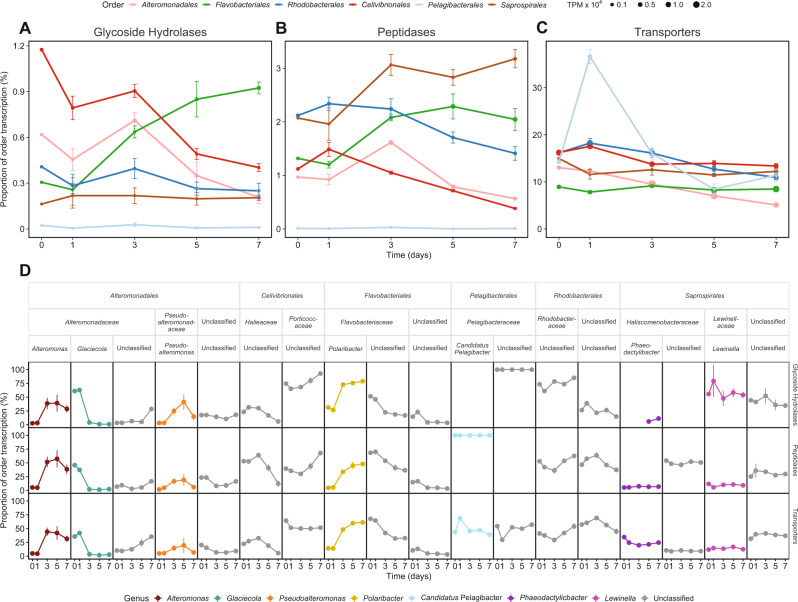
Expression of genes involved in dissolved organic matter utilization and nutrient uptake during the mesocosm experiment. **A** Glycoside hydrolases (GHs), (**B**) peptidases (PEPs), and (**C**) transporters (TPs). Data for the six transcriptionally most active bacterial orders are shown as transcripts per million (TPMs; denoted by size of circles) and as order-normalized proportions of transcription (as percent of TPMs for each order). Error bars denote standard deviations (*n* = 3 mesocosms, except for day 0 where *n* = 1). **D** Relative order-normalized transcript abundances at the genus level for each of the studied target gene systems (i.e., GH, PEP, and TP).

Analysis of the transcribed genes involved in the utilization of phytoplankton-derived labile organic matter or nutrients showed taxon-specific transcription patterns throughout the phytoplankton bloom (Fig. 3). *Cellvibrionales* had the highest order-normalized levels of GH expression until day 3 (Fig. 3A, *y*-axis); and the highest relative expression of GHs, as normalized to the entire metatranscriptome, was recorded for *Alteromonadales* on day 3 (Fig. 3A, size of filled circles). These taxa were primarily represented by the *Porticoccaceae* family and the genera *Alteromonas* plus *Glaciecola*, respectively (Figs. 3D,  S14). In contrast, transcription of flavobacterial GHs increased three-fold during bloom progression to ~1% of order-normalized transcription on day 7 (Fig. 3A), mainly due to the genus *Polaribacter* (*Flavobacteriaceae*) (Figs. 3D,  S14). Also PEPs showed pronounced differences between bacterial taxa in both temporal expression dynamics and in relative transcription. For all orders, PEP transcription remained fairly constant during the bloom development phase (days 0 and 1), after which the transcriptional investment steadily increased in *Saprospirales* and *Flavobacteriales* (Fig. 3B), especially in *Polaribacter* (Figs. 3D,  S14). *Alteromonadales*, in turn, showed a pronounced peak in PEP expression on day 3 (Fig. 3B), due mainly to the genera *Alteromonas* and *Pseudoalteromonas* (Figs. 3D,  S14). *Rhodobacterales* generally had high relative PEP expression (reaching ~2.4%, Fig. 3B) compared to GHs (~0.3% of order-normalized transcription, Fig. 3A), and were primarily represented by unclassified *Rhodobacteraceae* (Figs. 3D,  S14). Membrane transporters (TPs) accounted for ~10–20% of order-normalized transcription in the studied bacteria (Fig. 3C). While *Cellvibrionales*, *Alteromonadales*, and *Flavobacteriales* primarily invested in the transcription of TonB-dependent transporters (TBDTs; on average accounting for around 40%, 30%, and 20% of their total TP transcription, respectively), *Rhodobacterales* and *Pelagibacterales* favored ABC-type transporter transcription (45–40% of TP transcription) (Fig. S15). The *Flavobacteriales* (e.g., *Polaribacter*) and *Saprospirales* (dominated by the genus *Phaeodactylibacter* and an unclassified taxon) depicted a relatively constant investment in transporters throughout the phytoplankton bloom (Figs. 3C, D, S14); note though that for *Flavobacteriales* this stability resulted from shifts between taxa within the *Flavobacteriaceae*, from”Unclassified” to *Polaribacter* (Fig. 3D). In the other orders, expression generally decreased one-fourth over time, although *Pelagibacteraceae* transporter expression increased ~3-fold during the bloom development phase (day 0 to 1), to a peak at 37% of order-normalized transcripts (dominated by *Candidatus* Pelagibacter, Figs. 3C, D, S14). Altogether, these results emphasize the broad range in temporal adjustments of transcriptional investment in DOM and nutrient scavenging processes that marine bacterioplankton perform during phytoplankton bloom progression.

### Transcription of DOM-utilization genes as putative drivers of functional succession during the mesocosm bloom

**Fig. 4:**
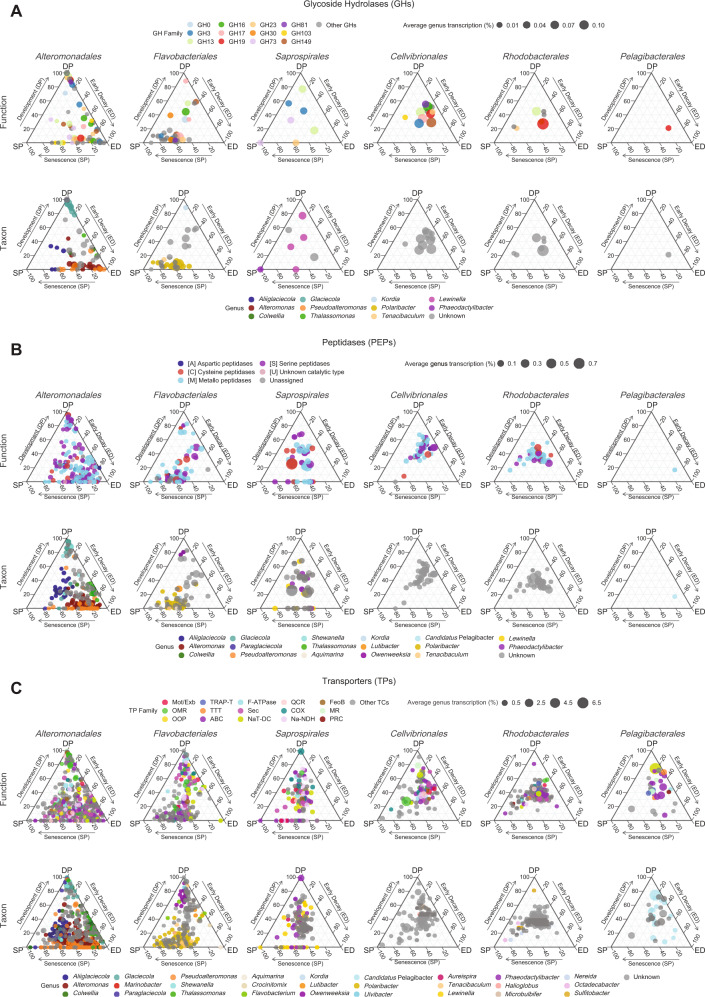
Ternary plots showing transcriptional differences between target gene systems (Function) and key bacterial taxa (Taxon) across mesocosm phytoplankton bloom phases. **A** Glycoside hydrolases (GHs), (**B**) peptidases (PEPs), and (**C**) transporters (TPs). Bloom phases are the development phase (DP, *n* = 4; days 0 and 1), early decay (ED, *n* = 3; day 3), and senescence phase (SP, *n* = 6; days 5 and 7). Bubble sizes denote normalized transcript abundances of individual GH families or PFAMs per genus averaged over all days in percent. The top 12 most abundant GH families are shown in color, all others in grey. All PEPs are color-coded according to their proteolytic families in the MEROPS database. The top 12 most abundant TP families in addition to TC TTT, MR, and PRC are shown in color. Abbreviation of TC families: Mot/Exb - The H^+^- or Na^+^-translocating Bacterial Flagellar Motor/ExbBD Outer Membrane Transport Energizer; OMR - Outer Membrane Receptor (here TonB-dependent transporters; TBDTs); OOP - OmpA-OmpF Porin; TRAP-T - Tripartite ATP-independent Periplasmic Transporter; TTT - Tricarboxylate Transporter; ABC - ATP-binding Cassette; F-ATPase - H^+^- or Na^+^-translocating F-type, V-type and A-type ATPase; Sec - General Secretory Pathway; NaT-DC - Na^+^-transporting Carboxylic Acid Decarboxylase; QCR - Proton-translocating Quinol:Cytochrome c Reductase; COX - Proton-translocating Cytochrome Oxidase; Na-NDH - Na^+^-translocating NADH:Quinone Dehydrogenase; FeoB - Ferrous Iron Uptake; MR - Ion-translocating Microbial Rhodopsin, PRC - Photosynthetic Reaction Center.

To further investigate the dynamics in bacterial substrate usage across bloom phases, we visualized the transcription of GHs, PEPs, and TPs in ternary plots (Fig. 4). We found pronounced differences between orders in grouping patterns resulting from: (i) differences in temporal expression of individual genes that were shared between orders (Fig. 4 and S15), and (ii) the expression of genes restricted to particular orders (or shared in different combinations; Fig. S16). Moreover, changes in GH, PEP, and TP transcription over time within orders, especially in the *Alteromonadales* and *Flavobacteriales*, resulted from changes in transcription ascribed to successional dynamics of different genera, as seen by comparing individual data points in the function plots to the corresponding taxon plots (Fig. 4). In the three transcribed gene systems, this was most evident as shifts in dominance in transcription from *Glaciecola* to e.g., *Alteromonas* and *Pseudoalteromonas* (in *Alteromonadales*) and from unclassified *Flavobacteriaceae* to *Polaribacter* (in *Flavobacteriales*).

Divergent grouping of transcribed GH genes affiliated with *Alteromonadales* and/or *Flavobacteriales* was observed along the early decay axis or toward the senescence phase axis, respectively (Fig. 4A), due to e.g., genes that likely code mostly for endo-acting laminarinases (e.g., GH17, GH16), exo-acting laminarinases, and α-amylases (GH3 and GH13, respectively) (Fig. 4 and S15). The other orders transcribed only few GHs (note *Saprospirales* GH3 and GH13 in the bloom development and early decay phases), or one as in *Pelagibacterales*. Sulfatase transcription was generally very low, with potential utilization of sulfated carbon compounds among *Planctomycetes*, *Rhizobiales*, and *Rhodobacteraceae* (Fig. S17).

Transcribed peptidase genes of *Alteromonadales* grouped primarily along the early decay axis (Fig. 4B), although not directly on the axis - indicating their transcription was maintained into the senescence phase to a higher degree than GHs. Differences over time were observed for diverse sets of metallo- (e.g., M23, M20, M41), serine- (e.g., S8, S9, and S24), and cysteine peptidases (e.g., C26). In contrast, expressed *Flavobacteriales* PEP genes, similar to their GHs, grouped away from the central part of the ternary plot toward the senescence axis (Fig. 4B). The relative transcriptional investment, in particular of intracellular cysteine peptidase C56, metallo- (e.g., M50, M20, and M1), and serine peptidases (e.g., S24 and S41) differed between *Flavobacteriales* and *Alteromonadales* (Fig. 4B and S15). The transcribed *Rhodobacterales* and *Cellvibrionales* PEP genes formed fairly tight clusters toward the center of the ternary plot, indicating that transcription of these genes remained largely stable during the bloom. Also *Saprospirales* showed a relatively stable investment, although several peptidases deviated (e.g., C14, M24, and M41) or were not expressed during the development phase (Fig. 4B and S15B).

Clustering of the many transcribed transporter genes affiliated with *Alteromonadales* (Fig. 4C) was in line with their overall strong response primarily during early decay (i.e., day 3; Figs. 1H, 2A). *Alteromonadales* were particularly active in transcribing TBDTs and general secretory pathway (Sec) family genes (Fig. S15). For *Flavobacteriales* transporters, there was a tight cluster centered in the ternary and a second cluster tightly aligning with the bloom senescence axis. The first cluster included secretory pathway and cation transport systems (e.g., 3.B.1 NaT-DC and 3.D.5 Na-NDH), whereas the second cluster was enriched in ABC-type transporters and the outer membrane factor (1.B.17 OMF). In contrast to the tight clustering of *Rhodobacterales* transporters (3.A.1 ABC for sugars, branched-chain amino acids, and DMSP), the spread of *Cellvibrionales* indicated a pronounced divergence in temporal expression of particular transporters. *Cellvibrionales* showed a high transcriptional investment in TBDTs (up to ~60% of their total TP transcription on day 7) and had the highest proteorhodopsin transcription during bloom development together with *Pelagibacterales* (Fig. S15C). In *Pelagibacterales*, the most abundant transporters were the Na^+^-transporting carboxylic acid decarboxylase (NaT-DC) that peaked on day 2 and the ABC Superfamily (Fig. 4C).

### Responsiveness of functional gene expression (GHs, PEPs, and TPs) in the field compared to mesocosms

**Fig. 5:**
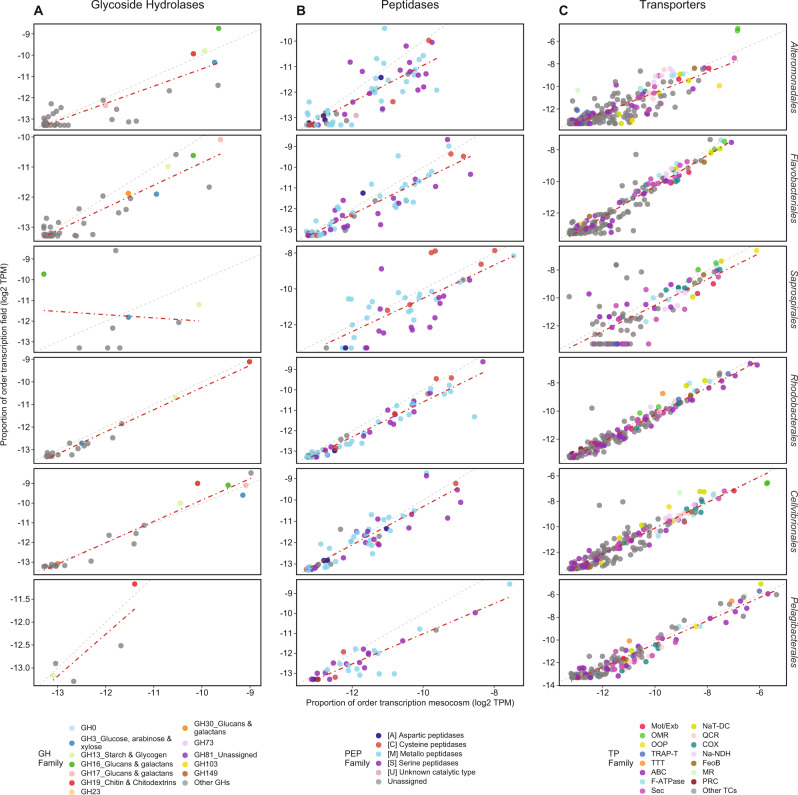
Comparison of order-specific gene transcription patterns between the mesocosms and the field on day 3. **A** Glycoside hydrolases (GHs), (**B**) peptidases (PEPs), and (**C**) transporters (TPs). The 1:1 line is shown in gray, whereas the red line shows a linear fit considering all target PFAMs. Color-legend same as Fig. 4. **A** Top 12 most abundant GH families, (**B**) all PEP classes, and in (**C**) top 12 most abundant TC families and TTT, MR, and PRC. Detailed information on gene annotations, transcripts per million (TPM) and percentage of order-specific transcription are given in Table S1 and linear regression statistics in Supplementary Material Tables S1 and S2.

Regression analyses showed that transcription of GHs, PEPs, and TPs were strongly and positively correlated between mesocosms and field samples, except for GHs in *Saprospirales* (Fig. S18, Supplemental Material Table S1). Overall, GHs and PEPs (Fig. S18A, B) showed some variability, with slopes between 0.5 and 1.2 and *R*^2^_*adj*_ values between 0.32 and 0.97, whereas TPs (Figs. S18C, S19) were similarly expressed (slopes: 0.9–1.1 and *R*^2^_*adj*_: 0.7–0.96). The early decay phase (day 3), during which major nutrient transitions occurred in the mesocosms, showed that GHs for the orders *Alteromonadales*, *Rhodobacterales*, and *Cellvibrionales* were highly correlated between mesocosms and the field (slopes > 0.8). Nevertheless, putative cellulases, α-amylase, exo- and endo-acting glucanases/laminarinases and chitinase (e.g., GH3, 13, 16, and 19) deviated from this pattern and were more responsive to nutritional conditions in the field. In contrast, *Flavobacteriales* showed a higher transcriptional investment in putative GHs in the mesocosms compared to the field (slopes < 0.8, Supplementary Material Table S2). These included e.g., GH3 and GH23, and exo-acting β-N-acetylglucosaminidases (GH20) targeting amino-sugars, and endo-β−1,4-mannanases (e.g., GH26) for hydrolyzing plant polysaccharides (Fig. 5A). In the field, there was a tendency to higher transcription of GHs involved in the degradation of structural polymers such as chitin and peptidoglycan (e.g., GH15, 73, 81, 144, 158) (Fig. 5A, S18). The negative correlation of GH transcription between mesocosm and field for *Saprospirales*, together with a tendency of higher transcription of PEPs and TPs in mesocosms, indicated a minor role of these *Bacteroidetes* in the upwelling (Figs. S18, S19).

For PEPs, most orders showed a fairly similar transcriptional investment in both systems (Fig. 5B; PEPs aligning with the 1:1 line). Still, *Alteromonadales* and *Pelagibacterales* tended to have higher expression in the mesocosms (Fig. 5B, slope < 1); note though those relationships changed on day 7 (Figs. S18B,  S19). Compared to GHs and PEPs, transporter expression was highly consistent between the mesocosms and field (slopes: 0.9–1) (Fig. 5C)—especially for *Flavobacteriales* and *Rhodobacterales* (slopes > 0.96). Inorganic phosphate transporters (PiTs) showed higher proportions in *Flavobacteriales* in the field, whereas ABC transporters were more important in mesocosms. *Pelagibacterales* showed the lowest number of regulated genes (slope ~1) (Fig. 5C, Supplementary Material Table S2), but an exceptional transcriptional investment in transporters during bloom development (Figs. 3C, S19).

**Fig. 6:**
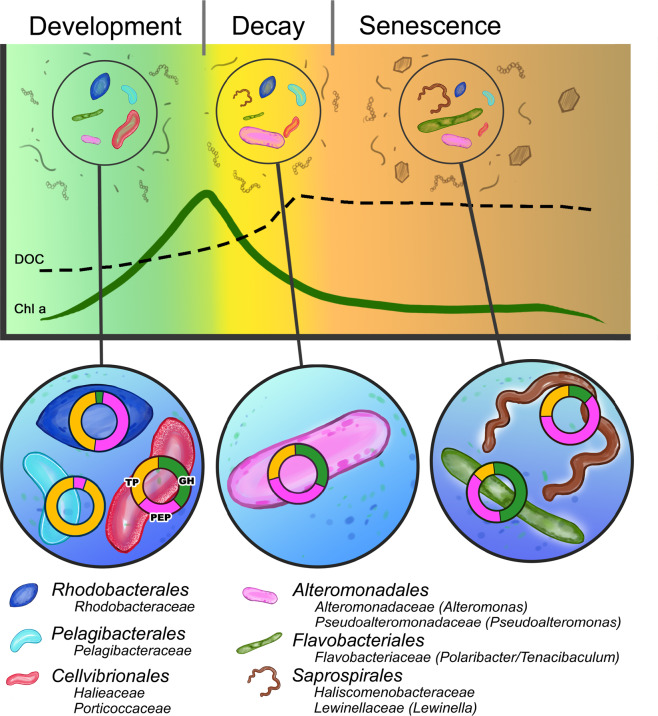
Conceptual model of order-specific transcription cascades across upwelling phytoplankton bloom phases. In the graph circles, cell sizes of the six studied bacterial orders denote their relative contribution to overall community transcription. Gray shapes outside the graph circles indicate tentative compositional changes in the dissolved organic matter pool. In the lower circles, the transcriptional response of the bacterial orders characteristic to each of the bloom phases is indicated. Doughnuts denote the transcription of gene systems for dissolved organic matter utilization and nutrient uptake: glycoside hydrolases (GHs), peptidases (PEPs), and transporters (TPs). The size of doughnut sections reflects differences in the relative allocation of transcriptional effort to the different gene systems.

We generated a conceptual model of the transcriptional cascades of major bacterial taxa across an upwelling bloom (Fig. 6) that builds on the genetic analyses in general and on the comparative analysis of order-specific gene transcription patterns of GHs, PEPs, and TPs between the mesocosms and the field in particular (Figs. 5 and  S18). The model outlines inferred changes in DOM and the identity of bacterial orders and families (and genera where possible) that dominate the transcription in different bloom phases.

## Discussion

### Transcriptional cascades across phytoplankton bloom phases

Our findings portray rapid adjustments in molecular mechanisms underlying functional traits of marine bacteria that can influence carbon cycling in upwelling waters. Since transcriptional responses in the mesocosms distinctly changed as a function of the phytoplankton bloom development phase, to the sharp peak in Chl *a* into the early decay and senescence phases, we here discuss responses of the studied bacteria according to bloom phases. Still, the taxonomic detail obtained from different genes or by different molecular approaches differed depending on the representation of taxa in current databases. Nevertheless, our analyses of 16S rRNA genes in community DNA and of total metatranscriptomes, including expressed targeted gene systems (i.e., GHs, PEPs, TPs, and STs) along with phylogenetic analyses of transcribed genes conserved across broad groups of bacteria (i.e., genes for RecA and ribosomal protein L12), provided a coherent view on the taxonomic identity of the key bacterial players in the studied upwelling bloom system.

The transcriptional dominance during bloom development of such phylogenetically different bacterial orders as *Pelagibacterales*, *Rhodobacterales*, and *Cellvibrionales* was noteworthy. The *Pelagibacterales* (SAR11 clade; here *Pelagibacteraceae*) are adapted to oligotrophic conditions, with streamlined genomes and fairly low transcriptional plasticity in combination with highly expressed high-affinity ABC transporter systems [55, 56]. In contrast, the *Rhodobacterales* (here mostly *Rhodobacteraceae* represented by the Roseobacter clade) life strategies range from streamlined oligotrophs to metabolically versatile opportunists [6, 57]. Yet, both *Pelagibacteraceae* and *Rhodobacteraceae* typically engage in the assimilation of phytoplankton-derived metabolites, in particular low-molecular-weight dissolved organic matter (LMW-DOM) [56, 58–61]. Knowledge of the ecophysiology of *Cellvibrionales* is limited, although they are common players in the global ocean [31, 62–64]. The order, in our study mainly represented by *Halieaceae* and *Porticoccaceae*, includes bacteria isolated from macroalgae or seaweed that possess agarolytic capabilities, such as *Agarilytica rhodophyticola* [65] and *Gilvimarinus polysaccharolyticus* [66]. Our results indicated that the *Cellvibrionales* families were similar to *Rhodobacteraceae* with respect to the number of expressed GHs and PEPs, but with substantially higher expression levels for the former. Moreover, *Cellvibrionales* dominated transcription of TBDTs, which together with their high transcription of GHs suggests a crucial role in the turnover of labile carbohydrates. As such, polysaccharide hydrolysis by different *Cellvibrionales* families potentially complements the *Rhodobacteraceae* and *Pelagibacteraceae* toward the turnover of LMW-DOM compounds.

A remarkable feature shared by the *Pelagibacteraceae*, *Rhodobacteraceae*, and *Cellvibrionales* families was their very limited transcriptional response to the strong DOC pulse on day 3. This could have been expected for pelagibacters, given their oligotrophic life strategy and their recognized specialization on LMW-DOM like carboxylic acids and DMSP [56, 67]. Nevertheless, *Pelagibacteraceae* were highly dynamic in their membrane transporter expression before the DOC pulse, focused on organic acids and DMSP (e.g., TTT, NaT-DC, TRAP-T, and ABC). However, the limited response by *Rhodobacteraceae* was surprising given that these bacteria (i.e., roseobacters such as *Planktomarina*) typically are referred to as dominant components of the bacterial community during phytoplankton blooms [6]. Incidentally, a lack of pronounced responses to phytoplankton decay DOM by both *Pelagibacteraceae* and *Rhodobacteraceae* was also observed across a spring bloom in the North Sea [11]. These findings and other studies suggest a limited involvement by *Rhodobacteraceae* in the degradation of high-molecular-weight DOM (HMW-DOM) compounds from massive phytoplankton decay [6]; in part due to being outcompeted by HMW-DOM specialists among e.g., *Gammaproteobacteria* and *Flavobacteriia* [68]. Our results provide the environmental context to model organism work showing *Rhodobacteraceae* as sensitive interaction partners that benefit from their proximity to active phytoplankton with which they can exchange metabolites [69–71].

An important feature of the early decay phase was the burst in *Alteromonadales* transcription, especially transcripts affiliated with the genera *Alteromonas* (*Alteromonadaceae*) and *Pseudoalteromonas* (*Pseudoalteromonadaceae*). These genera are widespread opportunists [27, 68, 72] and efficient scavengers of (algal) polysaccharides (e.g., laminarin, alginate, and pectin) [29, 30, 73, 74]. Beyond being opportunists in experiments, *Alteromonas* relatives are increasingly observed in various natural waters [74, 75]. Indeed, the pronounced transcriptional response in several GH genes showed that *Alteromonadales* rapidly exploited diverse polysaccharides or possibly glycoproteins. Notably though, this was combined with a broad set of PEPs, potentially related to the use of released proteins [76], and ABC transporters (e.g., for cobalamin) and outer membrane porins (potentially allowing surface attachment for utilization of polymers in aggregates or decaying phytoplankton). Altogether, our transcriptional analyses suggest that the success of *Alteromonadales*, and especially *Alteromonas* and *Pseudoalteromonas*, under bloom decay conditions fundamentally relies on the ability to exploit a palette of labile biopolymers, particularly polysaccharides and proteins [72, 76].

The early decay phase triggered a pronounced transcriptional response also by *Bacteroidetes*, which in the senescence phase further increased in relative transcription levels compared to *Alteromonadales*. Early bacterioplankton community composition studies showed that, in particular, members of the *Flavobacteriaceae* thrive upon phytoplankton bloom demise, suggesting a preference for HMW-DOM [77, 78]. Indeed, *Bacteroidetes* are efficient degraders of algal polysaccharides (e.g., laminarin, alpha-glucans, and sulfated xylans) [11, 79, 80] and proteins [29, 81–84]. These bacteria engaged little in transcription of sulfatases as compared to *Planctomycetes* and *Rhizobiales*. This is in line with previous reports showing that members of the *Plactomycetes* and *Verrucomicrobia* are specialized in the degradation of complex polysaccharides such as fucoidan [85–87]. Here, *Flavobacteriales* and *Alteromonadales* overall expressed a similar number of GH and PEP. In fact, they shared several GHs (e.g., GH3, 17, and 16) that accounted for similar proportions of their transcription, suggesting an important role of laminarin (a beta-1,3-glucan used for carbon storage in phytoplankton, particularly in diatoms), thus indicating an overlapping bacterial substrate range (i.e., niche space). However, Flavobacteriales showed a broader suite of expressed enzymes for hydrolyzing plant polysaccharides (endo-β-1,4-mannanases—e.g., GH26). While the source of mannans during algae blooms remains unclear, the cell walls of some diatoms contain mannans [88]. Thus, our findings suggest that certain *Flavobacteriales* mediate alpha- and beta-mannan degradation. *Alteromonadales*, in turn, transcribed higher levels of TBDTs and one-third more ABC-transporters (26 PFAMs) compared to *Flavobacteriales* and the temporal development of e.g., TBDT and porin genes differed substantially. These findings confirm previous observations on taxon-specific differences in transporter expression [11] and the importance of TBDTs for organic matter acquisition during phytoplankton blooms [15, 89], and provide novel mechanistic understanding on the divergent temporal evolution of hydrolytic enzyme and transporter transcription between the two taxa. In particular, the proportionately increased *Flavobacteriaceae* transcription of diverse enzymes toward bloom senescence suggested these bacteria are capable of exploiting a broader variety of phytoplankton-derived biopolymers.

### Interpreting bacterioplankton transcriptional responses to upwelling-driven blooms

The annual cycle of phytoplankton biomass in the NW Iberian upwelling system is characterized by a spring and summer bloom season representative of temperate shelf seas [90–92]. Rather than continuous blooms in each season, intermittent upwelling events induce variability in the duration of sequential bloom cycles from two to 20 days from initiation to complete dissipation [93–98]. The duration of the field and mesocosm phytoplankton blooms in our study (eight days) falls within this range, although the amplitude of changes in Chl *a* and DOC concentrations was more pronounced in the mesocosms. These differences are consistent with the higher initial nutrient concentrations in the mesocosms (intentionally obtained by water mass mixing) compared to the field, and the lack of loss factors such as advective or diffusive processes. It should also be noted that the mesocosm senescence phase was not observed in the field, likely due to the upwelling-driven injection of nutrients to the surface waters from day 4 onward. Nevertheless, the cascade of order-level transcriptional responses in gene systems for utilization of organic matter and nutrients that developed in parallel in the field and in the mesocosms was remarkably similar (except *Saprospirales* responses). Thus, it is pertinent to interpret the experimental results on resource utilization in the context of the natural settings.

What initially appeared a puzzling result was the substantial increase in *Cellvibrionales* transcription with bloom progression in the field but decrease in the mesocosm. Based on the generally high expression levels of GHs during mesocosm bloom development, along with the limited transcriptional response to the DOC pulse in the mesocosms on day 3, we propose that the *Cellvibrionales* families *Haliaceae* and *Porticoccaceae* largely rely on the utilization of polysaccharides released from physiologically deteriorating phytoplankton (but not from mass lysis). In contrast, the strong increase in transcription of recognized opportunist *Alteromonadales* genera like *Alteromonas* and *Pseudoalteromonas* [72, 99] in the mesocosms was less surprising. However, *Alteromonadales* accounted for up to 4% of the relative transcription in the field samples, as represented primarily by the genus *Glaciecola*, and for example *Alteromonas* is not uncommon in field studies of natural phytoplankton blooms [14], placing it as a potentially important player in upwelling systems. Our mesocosm findings suggest that in ecosystems with recurrent phytoplankton blooms, a diverse set of *Alteromonadales* genera are not merely opportunists, but rather, fine-tuned scavengers able to take advantage of labile biopolymers from lysing phytoplankton cells (selected polysaccharides and proteins; preferably supplied at a reasonably stable rate). These gammaproteobacterial taxa were accompanied by pelagibacters and roseobacters, which appear to be competitive in the quest for various labile LMW-DOM compounds under the transient high-nutrient conditions associated with phytoplankton blooms in upwelling systems (see details on these taxa above). Particularly, the pelagibacters are highly oligotrophic bacteria, yet some lineages of the SAR11 clade prefer coastal zones [56]. Lastly, we emphasize the recognized importance of *Flavobacteriaceae* in organic matter degradation during bloom senescence [11, 77], probably due to their combined ability to engage unusually diverse sets of enzyme systems for utilization for both polysaccharides and proteins [82]. For *Saprospirales*, we foresee that the high investment in peptidase transcription could provide an advantage over *Flavobacteriaceae* upon massive release of protein from phytoplankton lysis, resulting in resource partitioning of proteins between distinct *Bacteroidetes* taxa.

Our transcriptomics results on resource partitioning suggest that the chemical characteristics of important components of the DOM pool rapidly change during phytoplankton bloom succession. Bacteria can contribute to such DOM remodeling through differences between bacterial groups in the demands for, or utilization efficiency of, key elements like carbon and nitrogen, as observed for roseobacters compared to *Bacteroidetes* [100]. Accordingly, adjustment of glycoside hydrolase and peptidase expression, and corresponding membrane transporters, to selectively target carbon from carbohydrates or nitrogen-rich compounds like proteins could influence DOM pool stoichiometry. Our findings also suggest that transcriptional analysis of metabolic plasticity in nutrient acquisition can provide novel knowledge of mechanisms that underlie bacterioplankton succession under upwelling conditions leading to phytoplankton blooms, and how this relates to the labile DOM pool that accounts for a large fraction of surface ocean carbon fluxes [3, 23]. Given coastal zones contribute disproportionately to ocean productivity [1], rapid dynamics in bloom progression and bacterial responses would ultimately shape the biogeochemistry of the contemporary ocean. These lines of reasoning indicate that uncovering the linkages between bacterial activity and spatiotemporal variability in DOM chemical composition represents a tangible pursuit for microbial oceanography.

## Supplementary information

Supplemental Material

Figure S1

Figure S2

Figure S3

Figure S4

Figure S5

Figure S6

Figure S7

Figure S8

Figure S9

Figure S10

Figure S11

Figure S12

Figure S13

Figure S14

Figure S15

Figure S16

Figure S17

Figure S18

Figure S19

Table S1

Supplemental Material Table S1

Supplemental Material Table S2

## Data Availability

Ribosomal RNA gene sequence data have been deposited in the European Nucleotide Archive (ENA) at EMBL-EBI (https://www.ebi.ac.uk/ena), under project accession numbers PRJEB36188 (16S rRNA gene) and PRJEB36099 (18S rRNA gene). Metatranscriptome sequences are available at the EMBL-EBI European Nucleotide Archive repository (https://www.ebi.ac.uk/ena), under the project accession PRJEB36727 (mesocosms; ERS5512667-ERS5512693) and PRJEB36728 (field samples; ERS5513557-ERS5513582).
