# Supplementary material for: Rapid bacterioplankton transcription cascades regulate organic matter utilization during phytoplankton bloom progression in a coastal upwelling system
Source: ISME J. 2022 Jul 8;16(10):2360–72. doi: 10.1038/s41396-022-01273-0 (PMC9478159; doi:10.1038/s41396-022-01273-0)
Supplement: Supplementary file 1 — Supplemental Material [file 41396_2022_1273_MOESM1_ESM.docx]

# Supplementary Material

**Rapid bacterioplankton transcription cascades regulate organic matter utilization during phytoplankton bloom progression in a coastal upwelling system**

Benjamin Pontiller, Sandra Martínez-García, Vanessa Joglar, Dennis Amnebrink, Clara Pérez Martínez, José M. González, Daniel Lundin, Eva Teira, Jarone Pinhassi

**Supplementary Methods**

*Study site, sampling, and experimental setup*

Seawater for this study was collected during the ENVISION cruise III (CTM2014-59031-P, PI: Eva Teira, [1]) onboard the R/V *Ramón Margalef* at station 3 (Stn 3) (42° 7' 42.3984'' N, 8° 55' 44.9724'' W) on August 6^th^, 2016 (Fig. S1). Water was collected with 10L Niskin bottles mounted on a rosette sampler (equipped with CTD sensors) from 20 m depth (152 L; derived from recent upwelling) and water from 5 m (38 L) with an early phytoplankton bloom microbial community. The pooled water was pre-filtered through a 200 µm filter net and three 208 L low-density polyethylene (LDPE) tanks were filled with 190 L (152L + 38L) of pooled water. Mesocosms were incubated onboard in a flow-through tank, continuously flushed with ambient water to ensure in situ water temperature, and exposed to *in situ* light conditions (i.e., light cycle and intensity). To prevent evaporation and contamination, tanks were covered with a transparent PCTE foil (102 µm thick). After sunset, tanks were covered with a light-tight lid until sunrise to prevent exposure to artificial light sources onboard. Tanks were stirred ~5 min prior to subsampling of ~5 L water that was used for standard rate measurements (e.g., bacterial abundance and production, chlorophyll *a*, and nutrients). DNA and RNA samples (~4 L each) were taken on days 0, 1, 3, 5, and 7, and the same days were sampled in the field at Stn 3 (5 m depth) (Fig. S1 and S2).

*Nutrient analysis*

Samples for inorganic nutrient analysis (NH_4_^+^, NO_2_^-^, NO_3_^-^, PO_4_^3-^, and SiO_4_^4−^) were collected in 50 mL polyethylene bottles and stored at –20°C until analysis by standard colorimetric methods with an Alpkem segmented flow analyzer [2]. Dissolved organic carbon (DOC) concentrations were obtained by filtering water through 0.2 μm filters (Pall, Supor) in an all-glass filtration system under elevated air pressure with N_2_. Filtered water was collected into pre-combusted (450°C, 12 h) 2 mL glass bottles, and immediately frozen at -20 ºC until analysis. After defrosting, samples were acidified with 150 µL of 25% (v/v) H_3_PO_4_ and measured with a Shimadzu TOC-V analyzer coupled in series with a TNM-1 chemiluminescence detector according to [3].

*Prokaryote abundance and heterotrophic production*

Bacterial heterotrophic production was estimated through the ^3^H-leucine incorporation method as described in [4] according to [5]. A 1:10 hot:total leucine solution was prepared (final concentration 1 μM leucine) and 1 mL of sample (in triplicates) were amended with 40 μL of the radioactive leucine solution (final leucine concentration 40 nM; 118 Ci mmol^-1^, Perkin Elmer) in 1.5 mL microtubes. Samples were incubated at *in situ* temperature in the dark for 1 h, and subsequently leucine incorporation terminated by spiking the samples with 50% TCA to a final concentration of 4.5%. In addition, one blank per treatment was prepared by inactivating the sample with 50% TCA (final concentration 4.5%) 15 minutes before leucine addition. Samples were stored at -20ºC until further analysis. For analysis, samples were thawed at room temperature (RT) and centrifuged for 10 min at 18ºC and 10000 g, washed with 1 mL of cold 5% TCA and centrifuged again (10 min, 10000 g at 18ºC). Finally, the pellet was resuspended in 1 mL liquid scintillation cocktail (FilterCount, Perkin Elmer) and incubated for ~ 24h at RT in the dark. The leucine incorporation rate was estimated by counting the radioactivity in a liquid scintillation counter (WALLAC mod. 1409-012) and subtracting the disintegration per minute (DPM) of the blank from the mean DPM counts of triplicate samples. Finally, heterotrophic bacterial production was calculated using the theoretical leucine to a carbon conversion factor of 3.1 kg C mol Leu^-1^ [5].

*Microbial community composition and sequence data accession*

Prokaryotic and Eukaryotic community composition was determined and analyzed as described in [1]. In brief, ~2 L of water samples were sequentially filtered through 3 μm-pore-size Nucleopore polycarbonate filters (Whatman) and 0.22 μm-pore-size Sterivex filters (GP 0.22 μm, EMD Millipore), immediately frozen in liquid nitrogen and preserved at -80 °C. DNA contained in particles retained on the 3 μm-pore-size filters was extracted by using the PowerSoil DNA isolation kit (MoBio Laboratories, CA, USA) and from the 0.2 μm-pore-size filters with the PowerWater DNA isolation kit (MoBio Laboratories), according to the manufacturer’s instructions. For prokaryotes, the V4 and V5 regions of the 16S rRNA gene were amplified using the universal primers (515F and 926R) [6] from the pore-size <3.0 and >0.2 µm. The eukaryotic 18S rRNA gene was amplified from both size fractions using the primers TAReuk454FWD1 and TAReukREV3, targeting the V4 region of the 18S rRNA gene [7]. Amplicons were sequenced on a MiSeq platform (Illumina, Inc.) to obtain 2×300 bp paired-end reads at the Research and Testing Laboratory (Lubbock, TX, USA). Prokaryotic (16S rRNA genes) raw amplicon reads were processed using the Ampliseq pipeline (v2.2.0) [8] that utilized FastQC (v0.11.9) [9], MultiQC (v1.9) [10], Cutadapt (v3.4) [11], QIIME2 (v2019.10.0) [12], and DADA2 (v1.14.1) [13]. Eukaryotic (18S rRNA genes) amplicon reads were processed with the R package DADA2 (v1.14.1) [13]. For 16S and 18S, forward and reverse raw reads were trimmed to 259 and 199 excluding primers, respectively. Trimmed forward and reverse reads were denoised (using derepFastq), read pairs merged (mergePairs), and bimeras removed (removeBimeraDenovo with a minimum abundance of parents = 8 and a minimum overabundance = 4). Taxonomic assignments of 16S rRNA gene amplicon sequence variants (ASVs) were made using the SILVA reference database (v138.1) [14]. Taxonomic assignments of 18S rRNA genes were made with the reference database PR2 [15] and the marine protist database from the BioMarKs [16] project. ASV tables of prokaryotes and eukaryotes were subsampled to the lowest number of reads (2080 and 1286, respectively), which were sufficient to describe prokaryotic and eukaryotic community compositions. A total of 484 unique ASVs of prokaryotes were identified. For eukaryotes, we combined the datasets derived from the 0.2 and the 3 μm-pore-size filters and normalized the reads from each filter fraction using the respective DNA yield as described in [17] and [18] (resulting in 1080 unique ASVs) because many eukaryotic ASVs were present in both size fractions (e.g., those having a cell size range including 3 μm). All sequence data have been deposited in the European Nucleotide Archive (ENA) at EMBL-EBI (https://www.ebi.ac.uk/ena), under project accession numbers PRJEB36188 (16S rRNA gene sequences) and PRJEB36099 (18S rRNA gene sequences).

*Metatranscriptomics analysis and sequence data accession*

For metatranscriptomics, ~3.5 L water was sequentially filtered through 3.0 µm-pore-size Nucleopore filters (Whatman) and Sterivex filter units (GP 0.22 μm, EMD Millipore), preserved in 2 mL RNAlater (Qiagen), immediately flash-frozen in liquid nitrogen and stored at -20˚C on board and later at -80˚C until further processing. Total RNA was extracted using a protocol adapted from [19] with RNeasy Mini Kit (Qiagen) as described in [20]. In brief, samples were DNase treated using TURBO DNA-free Kit (Invitrogen, Thermo Fisher Scientific). Samples were controlled for residual DNA by a 30-cycle PCR targeting the 16S rDNA gene with primers (27F and 1492R) including Milli-Q negative and *E. coli* DNA positive controls. Ribosomal RNA was depleted using RiboMinus Transcriptome Isolation Kit and RiboMinus Concentration Module (Thermo Fisher Scientific) and linearly amplified using the MessageAmp II-Bacteria RNA Amplification Kit (Thermo Fisher Scientific). Finally, TruSeq libraries were generated and sequenced at the Swedish National Genome Infrastructure, SciLifeLab Stockholm on a HiSeq 2500 platform (Illumina, Inc.) in rapid mode and with v3 chemistry to obtain 2×125 bp long paired-end reads. Detailed sequencing summary statistics are provided in Table S1.

The quality of individual paired-end reads was determined through FastQC [9] and MultiQC [10]. Illumina adapter sequences were removed with Cutadapt (v1.13) [11] with a maximum error rate of 0.1. Reads were trimmed with Sickle (v1.33) [21] in paired-end mode and Sanger quality values. Ribosomal RNA was filtered with ERNE (v2.1.1) [22] against an in-house database of stable RNA sequences from marine microbes. High quality reads were *de-novo* assembled with MEGAHIT (v1.1.2) [23] and default parameters. We ran individual assemblies (i.e., mesocosms and field station 3 separate). Open reading frames (ORFs) were determined with Prodigal (v2.6.3) [24] in single mode and default parameters for each assembly. The ORFs were subsequently clustered at a 99% level (most efficient cluster level that resulted in the lowest number of multiple matches and highest proportion of read alignment) with VSEARCH (v2.5.2) [25]. The nucleotide sequences of the cluster representative were translated into amino acid sequences with transeq from emboss (v6.6.0.0) prior to searching the NCBI Refseq non-redundant protein database (nt) [26] with DIAMOND (v0.9.24) [27]. Taxonomic annotations were assigned using MEGAN (v6.12.8) [28] with the additional longReads lcaAlgorithm (LCA) for assemblies. The MEGAN LCA approach is conservative and will assign a higher-level taxon to a sequence when lower ranks contain alignments of contradictory taxonomy. Reads were mapped with bowtie2 (v2.3.5.1) [29] in paired-end mode to the clustered file containing the ORFs from the assemblies to obtain transcript counts per gene with SAMtools (v1.9) [30]. Eukaryotic ORFs were omitted from downstream analyses; the total number of prokaryotic (bacterial plus archaeal) ORFs was 930772. The sequence coverage for ORFs belonging to genera identified in the six bacterial orders on which our study focused ranged between 5.2 - 56.5 reads per ORF averaged per genus in the samples from the mesocosms and between 4.2 - 132.2 reads per ORF averaged per genus in the samples from the field. All samples that were used for individual assemblies and subsequent clustering are available at the EMBL-EBI European Nucleotide Archive repository (https://www.ebi.ac.uk/ena), under the project accessions PRJEB36727 (mesocosms), PRJEB36728 (field samples), PRJEB36712 (addition experiment 0.22-µm), and PRJEB42359 (Addition experiment 3-µm).

DOM active genes, i.e., glycoside hydrolases (GHs), peptidases (PEPs), transporters (TPs), and sulfatases (STs), were detected and classified by running all Pfams (Pfam v33) with HMMER3 [31] and the gathering score recommended for each model. HMMs specific for GHs were retrieved from the Pfams database to detect and classify the peptides in families and subfamilies. In addition, GHs were classified by running run-dbcan (v2.0.11; [32]) against the dbCAN2 database (http://bcb.unl.edu/dbCAN2; release date: July 31, 2018; [33]) using three search tools with the following default thresholds: HMMER (*e* value = 1e^-15^, coverage = 0.35), DIAMOND (*e*value = 1e^-102^), and Hotpep (frequency = 2.6, hits = 6). Consensus CAZyme family classifications were filtered as follows: i) Open reading frames (ORFs) that were predicted with one tool were excluded, if their %identity was < 50% (DIAMOND), coverage < 0.5 (HMMER) or hits < 10 (Hotpep). ii) ORFs with positive hits from more than one prediction tool were ranked based on the number of CAZyme family classifications and their relative %identity (DIAMOND), %coverage (HMMER), or relative hits + frequency (Hotpep) across all prediction tools. The highest ranking CAZyme family classification was kept for downstream analyses. The Pfam list specific for PEPs contained those with hits to the PEP subunit sequences in the MEROPS database (https://www.ebi.ac.uk/merops; [34]). Similarly, TP peptides were detected and classified according to the PFAMs specific for each type of TP as in the Transporter Classification Database (http://www.tcdb.org; [35]). STs were detected and classified by running HMMER3 [31] with the Pfam specific for sulfatases (PF00884) with its gathering score and subsequent blast search of the 1618 peptides against the SulfAtlas (v1.1.3) (http://abims.sb-roscoff.fr/sulfatlas; [36]) database with a minimum bit-score of 50, a minimum percent identity of 35, and a minimum coverage of 60% of the alignment resulting in 1468 peptides with a hit to the SulfAtlas peptides.

Detailed phylogenetic analysis of taxa that represented the most active components of the community was carried out based on the expression of two phylogenetic marker genes coding for the ribosomal protein L12, since this one was found to be the most highly expressed ribosomal protein gene, and RecA, a single copy gene universally present in Bacteria. To quantify the expression of these two genes in the most active and abundant taxa, a reference phylogenetic tree was used in both cases. These reference phylogenetic trees contained the taxa that best represented the groups expressing these genes in the samples as described next. To construct the reference peptide tree, all the corresponding peptides were retrieved from the genomes in the MAR database [37] using TIGR02012 (RecA) and PF00542 (L12 ribosomal protein) running HMMER3 [31] with their suggested gathering score. A taxonomy was assigned to the genomes with the GTDB Toolkit (GTDB-Tk; [38]). A smaller set of representative sequences was extracted based on the blastp results between the peptides from the assembled sequences and each of the reference databases, using a minimum percent identity of 80 and a bit-score of 50. The phylogenetic trees with the set of representative sequences were predicted with IQ-TREE [39]. The taxonomic groups on the trees were labeled according to the representative sequences within each of the clusters, considering that their bootstrap values at the lowest nodes were above 70. The labels were of the lowest taxonomic rank that would encompass the sequences in each of the clusters. RecA and L12 peptides from the assembled peptide sequences were retrieved using the previous blastp, along a HMMER3 search with each of the HMM models. The resulting RecA and L12 peptides from the assembled sequences were placed on the reference trees using the packages PaPaRa [40], EPA-ng [41] and gappa [42]. The amino acid substitution model was predicted with IQ-TREE. The taxonomic assignments of the peptides in each group were confirmed after visualization of the placed sequences with iToL [43]. This way, a taxonomic label was assigned to the peptides placed within the representative groups.

*Statistics, normalizations and visualization*

Principal component analysis (PCA) was performed with the function *prcomp* from the *stats* (v4.1.0) R package [44] on ORFs with at least 5 cpm in more than 3 samples. First, zero raw counts were replaced with estimates with the *cmultRepl* function and CZM method from *zCompositions* (v1.3.4) [45]. Second, centered log ratios (clr) were computed with the *codaSeq.clr* function from the *CoDaSeq* (v0.99.3) [46] and Euclidean distances calculated with the *dist* function in *vegan* (2.5-7) [47]. Order-specific PCAs were performed as described above but for each order-level separately to favor gene regulation over growth. Given that measures of dissimilarities in functional gene expression responses of bacteria over time can be influenced by two distinct mechanisms that determine the relative abundance of gene transcripts [48, 49]. First, if bacterial taxa have different and fairly stable expression profiles, differential growth will be the primary determinant of changes in community gene expression. Second, if bacteria actively regulate expression, shifts in community expression can occur even without changes in the relative abundance of the particular taxa. In natural communities these two mechanisms are expected to have an effect in gene expression.

Redundancy analysis (RDA) was performed on the same input data as described for the PCA above (clr). Environmental variables were selected based on pairwise Pearson correlations coefficients <0.9 and variance inflation factors <10 [50]. The suitability of an RDA was tested prior to analysis (gradient length of < 3.5). The model consisting of the variables Chl *a* (0.2 µm size fraction), Chl *a* 3 (µm size fraction), NH_4_^+^, and DOC was significant (*R^2^_adj_*= ~68%), Monte Carlo permutation tests showed that i) both RDA axes were significant after Holm correction for multiple testing (*p_adj_* < 0.004) and ii) the variables Chl *a* (3 µm size fraction) (*p_adj_* = 0.004) and DOC (*p_adj_* = 0.045) were significant, explaining ~27% and ~11% of the variation in bacterial community transcription, respectively (Fig. S13).

Genus level information on transcription of the studied gene systems was obtained by grouping normalized transcript counts at the respective taxonomic genus level within an order and functional GH family or PFAM level, so that the sum of normalized transcripts for all genera within an order and functional level adds up to 100% per sample (Fig. 3D and Fig. S14). Note that Fig. 3D represents only a subset of genera, therefore, sums are <100 per order.

For visualization in Ternary plots [51], we grouped order-normalized transcript counts at the GH family or PFAM level into development phase (DP - mean of day 0 and day 1; n = 4), early decay (ED - mean of day 3; n = 3), and senescence phase (SP - mean of day 5 and 7; n = 6), and standardized the counts to equal row sums considering all genera within an order and target gene set (GH, PEP, and TP). This allowed us to simultaneously visualize “Function” and “Taxon” information (Fig. 4).

*Field versus mesocosm comparison*

To obtain additional insight of the dynamics of this coastal upwelling system, we compared the expression of GHs, PEPs and TPs at the PFAM level between parallel mesocosm and the field samples through linear regressions on log2-transformed normalized (order) transcripts between mesocosm and station 3 with the *lm* function in *stats* [44] and additional functions from the *tidyverse* (e.g., *purrr*, *dplyr*, *tidyr*) [52], *modelr*[53], and *broom* [54]. Assuming a constant expression of a given group of genes by a certain order in both environmental conditions (i.e., mesocosm or field) a 1:1 relationship can be expected where X is the normalized log2-transformed abundance in mesocosms and Y is the normalized log2-transformed abundance in the field. A disproportional change in expression would result in a deviation from the 1:1 relationship, for example, below the line (slope <1) if the expression is higher in mesocosms over the field and vice versa (slope >1). Therefore, groups of genes (at the GH family or PFAM level) that are in close proximity to the 1:1 line can be considered as “stable” given that physicochemical parameters differed substantially between mesocosm and field from day 2 onward (Fig. S2), whereas genes with a high deviation (large residuals) can be tentatively considered as “responsive” to changes in environmental conditions (i.e., increase in DOC) (Supplemental Material Table S1 and S2).

*Phylogenetic analysis and diversity estimates by the expression analysis of the ribosomal protein L12 and the single copy gene recA*

The 200 most highly transcribed ORFs encoding L12 peptides (out of 1159) and RecA (out of 1183), accounted for 89% and 54% of average L12 gene and *recA* transcription (across all mesocosm and field samples), respectively. Phylogenetic trees of subsets of these 200 L12 and RecA peptides were visualized separately for *Gammaproteobacteria*,*Alphaproteobacteria*, and *Bacteroidetes* with the functions *ggtree* and *gheatmap* from the R package *ggtree* (v3.0.4) [55] and *ape* (v5.5) [56]. Richness and Shannon diversity indices were estimated on all L12 and RecA ORFs with the R packages *breakaway* (v4.7.3) [57] and *DivNet* (0.3.7) [58].

###

**Supplementary References**

1. Joglar V, Prieto A, Barber-Lluch E, Hernández-Ruiz M, Fernández E, Teira E. Spatial and temporal variability in the response of phytoplankton and prokaryotes to B-vitamin amendments in an upwelling system. Biogeosciences. 2020;17(10):2807-23.

2. Hansen H, Grasshoff K. Automated chemical analysis. Methods of seawater analysis. 2nd ed: Verlag Chemie, Weinheim; 1983. p. 347-95.

3. Álvarez-Salgado XA, Miller AEJ. Simultaneous determination of dissolved organic carbon and total dissolved nitrogen in seawater by high temperature catalytic oxidation: conditions for precise shipboard measurements. Mar Chem. 1998;62(3-4):325-33.

4. Kirchman D, K'nees E, Hodson R. Leucine incorporation and its potential as a measure of protein synthesis by bacteria in natural aquatic systems. Appl Environ Microbiol. 1985;49(3):599-607.

5. Smith DC, Farooq A. A simple, economical method for measuring bacterial protein synthesis rates in seawater using ^3^H-leucine. Mar Microb Food Webs. 1992;6(2):107-14.

6. Parada AE, Needham DM, Fuhrman JA. Every base matters: assessing small subunit rRNA primers for marine microbiomes with mock communities, time series and global field samples. Environ Microbiol. 2016;18(5):1403-14.

7. Logares R, Sunagawa S, Salazar G, Cornejo-Castillo FM, Ferrera I, Sarmento H, et al. Metagenomic 16S rDNA Illumina tags are a powerful alternative to amplicon sequencing to explore diversity and structure of microbial communities. Environ Microbiol. 2014;16(9):2659-71.

8. Straub D, Blackwell N, Langarica-Fuentes A, Peltzer A, Nahnsen S, Kleindienst S. Interpretations of Environmental Microbial Community Studies Are Biased by the Selected 16S rRNA (Gene) Amplicon Sequencing Pipeline. Front Microbiol. 2020;11:1-18.

9. Andrews S. FastQC: A quality control tool for high throughput sequence data. Ann Rev Mar Sci. 2010;3:401-25.

10. Ewels P, Magnusson M, Lundin S, Kaller M. MultiQC: Summarize analysis results for multiple tools and samples in a single report. Bioinformatics. 2016;32(19):3047-8.

11. Martin M. Cutadapt removes adapter sequences from high-throughput sequencing reads. EMBnet. 2011;17(1):10-12.

12. Bolyen E, Rideout JR, Dillon MR, Bokulich NA, Abnet CC, Al-Ghalith GA, et al. Reproducible, interactive, scalable and extensible microbiome data science using QIIME 2. Nature Biotechnology. 2019;37(8):852-57.

13. Callahan BJ, McMurdie PJ, Rosen MJ, Han AW, Johnson AJ, Holmes SP. DADA2: High-resolution sample inference from Illumina amplicon data. Nat Methods. 2016;13(7):581-3.

14. Quast C, Pruesse E, Yilmaz P, Gerken J, Schweer T, Yarza P, et al. The SILVA ribosomal RNA gene database project: improved data processing and web-based tools. Nucleic Acids Res. 2013;41(Database issue):D590-6.

15. Guillou L, Bachar D, Audic S, Bass D, Berney C, Bittner L, et al. The protist ribosomal reference database (PR^2^): a catalog of unicellular eukaryote small sub-unit rRNA sequences with curated taxonomy. Nucleic Acids Res. 2013;41(Database issue):D597-604.

16. Massana R, Gobet A, Audic S, Bass D, Bittner L, Boutte C, et al. Marine protist diversity in European coastal waters and sediments as revealed by high-throughput sequencing. Environ Microbiol. 2015;17(10):4035-49.

17. Dupont CL, McCrow JP, Valas R, Moustafa A, Walworth N, Goodenough U, et al. Genomes and gene expression across light and productivity gradients in eastern subtropical Pacific microbial communities. ISME J. 2015;9(5):1076-92.

18. Hernández-Ruiz M, Barber-Lluch E, Prieto A, Álvarez-Salgado XA, Logares R, Teira E. Seasonal succession of small planktonic eukaryotes inhabiting surface waters of a coastal upwelling system. Environ Microbiol. 2018;20(8):2955-73.

19. Poretsky RS, Gifford S, Rinta-Kanto J, Vila-Costa M, Moran MA. Analyzing gene expression from marine microbial communities using environmental transcriptomics. J Vis Exp. 2009;24:1-6.

20. Pontiller B, Martínez-García S, Lundin D, Pinhassi J. Labile dissolved organic matter compound characteristics select for divergence in marine bacterial activity and transcription. Front Microbiol. 2020;11:588778.

21. Joshi NA, Fass JN. Sickle: A sliding-window, adaptive, quality-based trimming tool for FastQ files. 1.33 ed2011.

22. Del Fabbro C, Scalabrin S, Morgante M, Giorgi FM. An extensive evaluation of read trimming effects on Illumina NGS data analysis. PLoS One. 2013;8(12):1-13.

23. Li D, Liu CM, Luo R, Sadakane K, Lam TW. MEGAHIT: An ultra-fast single-node solution for large and complex metagenomics assembly via succinct de Bruijn graph. Bioinformatics. 2015;31(10):1674-6.

24. Hyatt D, Chen GL, Locascio PF, Land ML, Larimer FW, Hauser LJ. Prodigal: prokaryotic gene recognition and translation initiation site identification. BMC Bioinform. 2010;11:119.

25. Rognes T, Flouri T, Nichols B, Quince C, Mahe F. VSEARCH: a versatile open source tool for metagenomics. PeerJ. 2016;4:e2584.

26. O'Leary NA, Wright MW, Brister JR, Ciufo S, Haddad D, McVeigh R, et al. Reference sequence (RefSeq) database at NCBI: Current status, taxonomic expansion, and functional annotation. Nucleic Acids Res. 2016;44(D1):D733-45.

27. Buchfink B, Xie C, Huson DH. Fast and sensitive protein alignment using DIAMOND. Nat Methods. 2015;12(1):59-60.

28. Huson DH, Beier S, Flade I, Górska A, El-Hadidi M, Mitra S, et al. MEGAN community edition - interactive exploration and analysis of large-scale microbiome sequencing data. PLoS Comput Biol. 2016;12(6):1-12.

29. Langmead B, Salzberg SL. Fast gapped-read alignment with bowtie 2. Nat Methods. 2012;9(4):357-9.

30. Li H, Handsaker B, Wysoker A, Fennell T, Ruan J, Homer N, et al. The sequence alignment/map format and samtools. Bioinformatics. 2009;25(16):2078-9.

31. Eddy SR. A probabilistic model of local sequence alignment that simplifies statistical significance estimation. PLoS Comput Biol. 2008;4(5)(5):e1000069.

32. Huang L, Zhang H, Wu P, Entwistle S, Li X, Yohe T, et al. dbCAN-seq: a database of carbohydrate-active enzyme (CAZyme) sequence and annotation. Nucleic Acids Res. 2018;46(D1):D516-D21.

33. Zhang H, Yohe T, Huang L, Entwistle S, Wu P, Yang Z, et al. dbCAN2: A meta server for automated carbohydrate-active enzyme annotation. Nucleic Acids Res. 2018;46(W1):W95-W101.

34. Rawlings ND, Barrett AJ, Thomas PD, Huang X, Bateman A, Finn RD. The MEROPS database of proteolytic enzymes, their substrates and inhibitors in 2017 and a comparison with peptidases in the PANTHER database. Nucleic Acids Res. 2018;46(D1):D624-D32.

35. Saier MH, Jr., Tran CV, Barabote RD. TCDB: The transporter classification database for membrane transport protein analyses and information. Nucleic Acids Res. 2006;34(Database issue):D181-6.

36. Barbeyron T, Brillet-Gueguen L, Carre W, Carriere C, Caron C, Czjzek M, et al. Matching the diversity of sulfated biomolecules: creation of a classification database for sulfatases reflecting their substrate specificity. PLoS One. 2016;11(10):e0164846.

37. Klemetsen T, Raknes IA, Fu J, Agafonov A, Balasundaram SV, Tartari G, et al. The MAR databases: Development and implementation of databases specific for marine metagenomics. Nucleic Acids Res. 2018;46(D1):D692-D99.

38. Chaumeil PA, Mussig AJ, Hugenholtz P, Parks DH. GTDB-Tk: a toolkit to classify genomes with the genome taxonomy database. Bioinformatics. 2019.

39. Minh BQ, Schmidt HA, Chernomor O, Schrempf D, Woodhams MD, von Haeseler A, et al. IQ-TREE 2: New models and efficient methods for phylogenetic inference in the genomic era. Mol Biol Evol. 2020;37(5):1530-34.

40. Berger SA, Stamatakis A. Aligning short reads to reference alignments and trees. Bioinformatics. 2011;27(15):2068-75.

41. Barbera P, Kozlov AM, Czech L, Morel B, Darriba D, Flouri T, et al. EPA-ng: Massively parallel evolutionary placement of genetic sequences. Syst Biol. 2019;68(2):365-69.

42. Czech L, Barbera P, Stamatakis A. Genesis and gappa: Processing, analyzing and visualizing phylogenetic (placement) data. Bioinformatics. 2020;36(10):3263-65.

43. Letunic I, Bork P. Interactive tree of life (iTOL) v5: An online tool for phylogenetic tree display and annotation. Nucleic Acids Res. 2021.

44. R Core Team. R: A language and environment for statistical computing. 4.1.0 ed. Vienna, Austria: R Foundation for Statistical Computing; 2021.

45. Palarea-Albaladejo J, Martín-Fernández JA. zCompositions — R package for multivariate imputation of left-censored data under a compositional approach. Chemometr Intell Lab Syst. 2015;143:85-96.

46. Gloor GB, Wu JR, Pawlowsky-Glahn V, Egozcue JJ. It's all relative: analyzing microbiome data as compositions. Ann Epidemiol. 2016;26(5):322-9.

47. Oksanen J, Blanchet FG, Friendly M, Kindt R, Legendre P, McGlinn D, et al. Vegan: Community ecology package. 2.5-5 ed2019.

48. Satinsky BM, Crump BC, Smith CB, Sharma S, Zielinski BL, Doherty M, et al. Microspatial gene expression patterns in the Amazon River plume. Proc Natl Acad Sci U S A. 2014;111(30):11085-90.

49. Salazar G, Paoli L, Alberti A, Huerta-Cepas J, Ruscheweyh HJ, Cuenca M, et al. Gene expression changes and community turnover differentially shape the global ocean metatranscriptome. Cell. 2019;179(5):1068-83 e21.

50. Borcard D, Gillet F, Legendre P. Numerical ecology with R. New York: Springer.; 2011.

51. Hamilton NE, Ferry M. ggtern: Ternary Diagrams Using ggplot2. J Stat Softw. 2018;87(Cn3):1-17.

52. Wickham H. Tidyverse: Easily install and load the ‘tidyverse'. 1.2.1 ed2017.

53. Wickham H. modelr: modelling functions that work with the pipe. 0.1.8 ed2020.

54. Robinson D, Hayes A, Couch S. broom: convert statistical objects into tidy tibbles. 0.7.0 ed2020.

55. Yu G, Smith DK, Zhu H, Guan Y, Lam TTY, McInerny G. ggtree: an r package for visualization and annotation of phylogenetic trees with their covariates and other associated data. Methods Ecol Evol. 2016;8(1):28-36.

56. Paradis E, Schliep K. ape 5.0: an environment for modern phylogenetics and evolutionary analyses in R. Bioinformatics. 2019;35(3):526-28.

57. Willis A, Martin BD, Trinh P, Teichman S, Barger K, Bunge J. breakaway: Species Richness Estimation and Modeling. 4.7.3. ed2020.

58. Willis AD, Martin BD. Estimating diversity in networked ecological communities. Biostatistics. 2020.

**Supplementary Figure legends**

**Figure S1**. Location and satellite imagery of the northeast Atlantic Ocean study area. (**A**) Geographical location of the study site, off the northwest Iberian coastline (Spain) and the coastal station (Stn 3). The color bar shows the sea surface temperature (SST) on August 5^th^, 2016 (day 0 of the mesocosm experiment). (**B**) Weekly chlorophyll *a* (Chl *a*) data covering the period two weeks before and after the start of the mesocosm experiment. (**C**) Chl *a* fluorescence (red-colored circles) and the sea surface temperature (SST, orange-colored circles) during the same period at Stn 3. Multi-scale Ultra-high Resolution (MUR) SST (fv04.1, Global, 0.01°) and Visible Infrared Imaging Radiometer Suite (VIIRS) provided by the National and Oceanic and Atmospheric Administration (NOAA) ERDDAP.

**Figure S2**. Nutrient dynamics during the phytoplankton bloom in the field and mesocosm samples over 8 days. Depicted are averages ± standard deviation (SD) of biological triplicates for mesocosm and single measurements of field samples from station 3 (Stn 3).

**Figure S3.** Microbial community composition during the coastal upwelling phytoplankton bloom and experimental mesocosm study. (**A**) Relative abundance of eukaryotic taxa as derived through 18S rRNA gene sequencing of the 3.0 and 0.22 μm-pore-size fraction. (**B**) Relative abundance of prokaryotic taxa as derived from 16S rRNA gene amplicon sequencing of the 3.0-0.22 μm-pore-size fraction. Note that no data is available for day 0 and day 5 replicates M1 and M2.

**Figure S4**. Simplified phylogenetic tree of *Gammaproteobacteria* based on a subset of the 200 most actively transcribed genes encoding the L12 ribosomal protein. A selected representation of reference sequences is shown. The heatmap shows the relative log-transformed transcripts per million (TPM) of individual L12 open reading frames (ORFs) in the field and in the experimental mesocosm.

**Figure S5**. Simplified phylogenetic tree of *Gammaproteobacteria* based on a subset of the 200 most actively transcribed genes encoding the RecA protein. To reduce the number of taxa on the tree, the figure shows a selected representation of reference sequences. The heatmap indicates the relative log-transformed transcripts per million (TPM) of individual RecA open reading frames (ORFs) in the field and in the experimental mesocosm.

**Figure S6**. Simplified phylogenetic tree of *Bacteroidetes* based on a subset of the 200 most actively transcribed genes encoding the L12 protein. The number of reference sequences has been reduced for simplicity. The heatmap shows the relative log-transformed transcripts per million (TPM) of individual L12 open reading frames (ORFs) in the field and in the experimental mesocosm.

**Figure S7**. Simplified phylogenetic tree of *Bacteroidetes* based on a subset of the 200 most actively transcribed genes encoding the RecA protein. Only a selection of reference sequences is shown. The heatmap shows the relative log-transformed transcripts per million (TPM) of individual RecA open reading frames (ORFs) in the field and in the experimental mesocosm.

**Figure S8**. Simplified phylogenetic tree of *Alphaproteobacteria* based on a subset of the 200 most actively transcribed genes encoding the L12 protein. To simplify the figure, the number of reference sequences was reduced. The heatmap shows the relative log-transformed transcripts per million (TPM) of individual L12 open reading frames (ORFs) in the field and in the experimental mesocosm.

**Figure S9**. Simplified phylogenetic tree of *Alphaproteobacteria* based on a subset of the 200 most actively transcribed genes encoding the RecA protein. A selected representation of reference sequences is shown to simplify the figure. Heatmap shows the relative log-transformed transcripts per million (TPM) of individual RecA open reading frames (ORFs) in the field and in the experimental mesocosm.

**Figure S10.** Principal component analysis (PCA) of prokaryotic community transcription in mesocosms and the field station 3 (Stn 3). (**A**) All genes and (**B**) Target genes (i.e., GH, PEP and TP). Analysis is based on quality filtered ORFs and centered log-ratio transformed counts (see Supplementary Material).

**Figure S11**. Diversity estimates of the ribosomal protein L12 gene expression and the single copy gene *recA* expression. (**A**) shows the richness and (**B**) the Shannon diversity of the L12 protein gene; (**C**) depicts the richness and (**D**) the Shannon diversity for the *recA* gene expression. Error bars and bubble size denote standard errors of the estimate.

**Figure S12**. Variance partitioning of the environmental variables chlorophyll *a* (3.0- and 3.0-0.2-µm-pore-size fractions), dissolved organic carbon (DOC), and ammonium (NH_4_^+^) used in the redundancy analysis (RDA) shown in Fig 2B.

**Figure S13**. Principal component analysis (PCA) of the most active orders in the mesocosms based on open reading frames (ORFs) which passed the filter step (>5 cpm in at least 3 samples), based on clr-transformed raw counts and Euclidean distances. Panels depict the temporal development of transcription profiles of (**A**) *Alteromonadales*, (**B**) *Flavobacteriales*, (**C**) *Saprospirales*, (**D**) *Rhodobacterales*, (**E**) *Cellvibrionales*, and (**F**) *Pelagibacterales*, during five different time points. Convex hulls are grouping the respective biological mesocosm replicates.

**Figure S14**. Relative transcription of glycoside hydrolases (GH), peptidases (PEP), and transporters (TP) from order to genus level. Depicted are relative transcript abundances of open reading frames (ORFs) at the genus level normalized to the total order transcription for each of the studied target gene systems (i.e., GH, PEP, and TP).

**Figure S15**. Heatmaps depicting log2-transformed relative abundances of transcripts normalized per order-level. (**A**) Glycoside Hydrolases - GHs; (**B**) Peptidases - PEPs; and (**C**) Transporters - TPs. Note that putative substrates were assigned to the most abundant GHs families when possible. PEPs were grouped into catalytic types based on the nomenclature used in the MEROPS peptidase database. TPs were grouped into families based on TP classification (TC) numbers and associated descriptive names depicted in abbreviated form: Mot/Exb - H^+^- or Na^+^-translocating Bacterial Flagellar Motor/ExbBD Outer Membrane Transport Energizer (Mot/Exb); OMR - Outer Membrane Receptor (here TonB-dependent transporters; TBDTs); OOP - OmpA-OmpF Porin; TRAP-T - Tripartite ATP-independent Periplasmic Transporter; TTT - Tricarboxylate Transporter; ABC - ATP-binding Cassette; F-ATPase - H^+^- or Na^+^-translocating F-type, V-type and A-type ATPase; Sec - General Secretory Pathway; NaT-DC - Na^+^-transporting Carboxylic Acid Decarboxylase; QCR - Proton-translocating Quinol:Cytochrome c Reductase; COX - Proton-translocating Cytochrome Oxidase; Na-NDH - Na^+^-translocating NADH:Quinone Dehydrogenase; FeoB - Ferrous Iron Uptake, MR - Ion-translocating Microbial Rhodopsin, PCR - Photosynthetic Reaction Center.

**Figure S16.** Overview of the number of unique and shared glycoside hydrolase families (GH families), peptidase (PEP), and transporter (TP) PFAMs among the six transcriptionally most active bacterial orders. The y-axis shows the number of GH families and PFAMs distributed among various combinations of bacterial orders (x-axis) based on presence/absence of transcribed genes in the studied taxa.

**Figure S17**. Transcription of sulfatases during the experimental mesocosm phytoplankton bloom. (**A**) Order-normalized transcript abundance of sulfatases. (**B**) Relative transcript abundances of sulfatases at the genus level normalized to the total order transcription of sulfatases.

**Figure S18.** Linear regressions of log2-transformed order-normalized transcript abundance (log2 TPM) between field and mesocosms for: (**A**) Glycoside Hydrolases - GHs; (**B**) Peptidases - PEPs; and (**C**) Transporters - TPs, for the days 1, 3, 5, and 7. The 1:1 dotted reference line is shown in gray, whereas the red dash-dotted line shows a linear fit considering all target PFAMs. Colors denote the twelve most abundant GH families, all PEP classes, and the top twelve most abundant TC families including TTT, MR, and PRC based on their mean normalized order-normalized transcription. Abbreviation of TC families: Mot/Exb - H^+^- or Na^+^-translocating Bacterial Flagellar Motor/ExbBD Outer Membrane Transport Energizer; OMR - Outer Membrane Receptor (here TonB-dependent transporters; TBDTs); OOP - OmpA-OmpF Porin; TRAP-T - Tripartite ATP-independent Periplasmic Transporter; TTT - The Tricarboxylate Transporter; ABC - The ATP-binding Cassette; F-ATPase - H^+^- or Na^+^-translocating F-type, V-type and A-type ATPase; Sec - General Secretory Pathway; NaT-DC - Na^+^-transporting Carboxylic Acid Decarboxylase; QCR - The Proton-translocating Quinol:Cytochrome c Reductase; COX - Proton-translocating Cytochrome Oxidase; Na-NDH - Na^+^-translocating NADH:Quinone Dehydrogenase; FeoB - Ferrous Iron Uptake; MR - Ion-translocating Microbial Rhodopsin, PCR - Photosynthetic Reaction Center.

**Figure S19.** Summary of slopes and standard deviation of residuals derived from linear regressions between log2-order-normalized transcripts in mesocosm versus field (Stn 3) as shown in Figure S18. A slope of 1 indicates that transcription in mesocosm and the field was the same, a deviation of slope <1 indicates that transcription was higher in mesocosms (gray area), whereas slopes >1 (white area) show the opposite. Depicted are slopes with a significant (*p* <0.05) linear regression fit. The size of the points corresponds to the standard deviation of the residuals (GH families or PFAMs) from the linear regression fit.

#####

**Supplemental Table legend**

**Table S1.** Metatranscriptome data summary for field and mesocosm samples.

**Supplemental Material Table legends**

**Supplemental Material Table S1.** Data tables containing additional information of expressed glycoside hydrolases - GH (1^st^ tab), peptidases - PEPs (2^nd^ tab), and transporters - TPs (3^rd^ tab). Abbreviation: Transcripts per million - TPM, Normalized transcripts per order-level - prct_of_order_transcription, The number of ORFs per PFAM - nr_orfs.

**Supplemental Material Table S2.** Summary statistics (residuals, coefficients, and model performances) of the linear regression models of order transcription in mesocosms and the field for glycoside hydrolases (GH), peptidases (PEP), and transporters (TP). Abbreviation: id - DOM active gene systems, pred - predicted values, resid - residual, regulation - if the residual of a given GH family or PFAM was within 10% of the maximum residual for a given gene system, day and order it was labeled as “responsive” otherwise as “constitutive”.
