# Supplementary material for: Rapid bacterioplankton transcription cascades regulate organic matter utilization during phytoplankton bloom progression in a coastal upwelling system
Source: ISME J. 2022 Jul 8;16(10):2360–72. doi: 10.1038/s41396-022-01273-0 (PMC9478159; doi:10.1038/s41396-022-01273-0)
Supplement: Supplementary file 21 — Table S1 [file 41396_2022_1273_MOESM21_ESM.docx]

| **TABLE S1** \| Metatranscriptome data summaries for field and mesocosm samples | | | | | | | | | | | | | | | | | | | | | | | | | | |  |  |  |  |
| --- | --- | --- | --- | --- | --- | --- | --- | --- | --- | --- | --- | --- | --- | --- | --- | --- | --- | --- | --- | --- | --- | --- | --- | --- | --- | --- | --- | --- | --- | --- |
| **Origin** |  | **Field** | | | |  |  |  | |  | |  | | **Mesocosm** | | | | | | | | | | | | | | |  |  |
| **Day** |  | **1** | **3** | **5** | **7** |  | **0** |  | **1** | | | | | |  | **3** | | |  | **5** | | | |  | **7** | | | | |  |
| **Replicate** |  |  | | | |  | **0** |  | **1** | | **2** | | **3** | |  | **1** | **2** | **3** |  | **1** | **2** | | **3** |  | **1** | **2** | | **3** | |  |
|  |  |  |  |  |  |  |  |  |  | |  | |  | |  |  |  |  |  |  |  | |  |  |  |  | |  | |  |
| # of sequenced raw reads $\times$ 10^6^ |  | 73.7 | 82.6 | 76.6 | 71.9 |  | 63.5 |  | 57.7 | | 69.2 | | 54.7 | |  | 65.4 | 77.7 | 63.9 |  | 71.4 | 64.5 | | 62.5 |  | 80.1 | 67.7 | | 69.4 | |  |
|  |  |  |  |  |  |  |  |  |  | |  | |  | |  |  |  |  |  |  |  | |  |  |  |  | |  | |  |
| % of rRNA |  | 63.1 | 65.0 | 63.5 | 64.5 |  | 61.6 |  | 62.5 | | 59.6 | | 60.6 | |  | 57.9 | 57.2 | 60.3 |  | 60.8 | 64.5 | | 64.3 |  | 58.9 | 59.0 | | 61.4 | |  |
|  |  |  |  |  |  |  |  |  |  | |  | |  | |  |  |  |  |  |  |  | |  |  |  |  | |  | |  |
| # of reads retained post rRNA depletion $\times$ 10^6^ |  | 26.7 | 28.4 | 27.4 | 25.0 |  | 23.9 |  | 21.3 | | 27.5 | | 21.2 | |  | 27.1 | 32.8 | 25.0 |  | 27.5 | 22.4 | | 21.9 |  | 32.4 | 27.3 | | 26.3 | |  |
|  |  |  |  |  |  |  |  |  |  | |  | |  | |  |  |  |  |  |  |  | |  |  |  |  | |  | |  |
|  |  |  |  |  |  |  |  |  |  | |  | |  | |  |  |  |  |  |  |  | |  |  |  |  | |  | |  |
| # of reads mapped to ORFs* $\times$ 10^6^ |  | 15.1 | 16.9 | 16.2 | 15.5 |  | 14.5 |  | 12.0 | | 16.4 | | 12.2 | |  | 16.9 | 20.7 | 15.6 |  | 15.1 | 11.9 | | 11.5 |  | 16.6 | 14.0 | | 12.9 | |  |
|  |  |  |  |  |  |  |  |  |  | |  | |  | |  |  |  |  |  |  |  | |  |  |  |  | |  | |  |
| % of reads annotated as Eukaryotic |  | 20.5 | 18.0 | 19.9 | 17.6 |  | 16.6 |  | 16.5 | | 17.4 | | 17.1 | |  | 13.8 | 12.3 | 12.9 |  | 25.8 | 24.5 | | 27.1 |  | 31.9 | 30.6 | | 31.8 | |  |
|  |  |  |  |  |  |  |  |  |  | |  | |  | |  |  |  |  |  |  |  | |  |  |  |  | |  | |  |
| % of reads annotated as Bacteria |  | 37.4 | 40.1 | 26.2 | 35.9 |  | 32.0 |  | 37.6 | | 35.5 | | 35.4 | |  | 50.7 | 53.8 | 55.4 |  | 25.8 | 27.9 | | 27.2 |  | 21.8 | 21.8 | | 21.3 | |  |
|  |  |  |  |  |  |  |  |  |  | |  | |  | |  |  |  |  |  |  |  | |  |  |  |  | |  | |  |
| % of reads annotated as Archaea |  | 0.7 | 0.7 | 1.6 | 2.2 |  | 3.3 |  | 1.6 | | 1.1 | | 1.3 | |  | 0.1 | 0.1 | 0.2 |  | 0.0 | 0.0 | | 0.0 |  | 0.0 | 0.0 | | 0.0 | |  |
|  |  |  |  |  |  |  |  |  |  | |  | |  | |  |  |  |  |  |  |  | |  |  |  |  | |  | |  |
|  |  |  |  |  |  |  |  |  |  | |  | |  | |  |  |  |  |  |  |  | |  |  |  |  | |  | |  |
| # of reads annotated as Bacteria and  Archaea $\times$ 10^6^ |  | 5.8 | 6.9 | 3.1 | 5.9 |  | 5.1 |  | 4.7 | | 6.0 | | 4.5 | |  | 8.6 | 11.2 | 8.7 |  | 3.9 | 3.3 | | 3.1 |  | 3.6 | 3.0 | | 2.8 | |  |
|  |  |  |  |  |  |  |  |  |  | |  | |  | |  |  |  |  |  |  |  | |  |  |  |  | |  | |  |
| % of which are Transporters |  | 14.5 | 15.0 | 14.3 | 17.2 |  | 15.7 |  | 16.9 | | 17.6 | | 17.2 | |  | 15.3 | 15.2 | 15.2 |  | 11.3 | 11.3 | | 11.2 |  | 11.8 | 11.3 | | 11.6 | |  |
|  |  |  |  |  |  |  |  |  |  | |  | |  | |  |  |  |  |  |  |  | |  |  |  |  | |  | |  |
| % of which are Peptidases |  | 2.3 | 2.5 | 2.4 | 2.4 |  | 2.4 |  | 2.7 | | 2.4 | | 2.7 | |  | 3.2 | 3.2 | 3.0 |  | 2.3 | 2.5 | | 2.3 |  | 2.4 | 2.1 | | 2.4 | |  |
|  |  |  |  |  |  |  |  |  |  | |  | |  | |  |  |  |  |  |  |  | |  |  |  |  | |  | |  |
| % of which are Glycoside hydrolases |  | 1.1 | 1.6 | 0.8 | 0.6 |  | 1.0 |  | 0.8 | | 0.7 | | 0.7 | |  | 1.3 | 1.3 | 1.3 |  | 0.8 | 0.9 | | 0.8 |  | 1.0 | 0.8 | | 0.9 | |  |
|  |  |  |  |  |  |  |  |  |  | |  | |  | |  |  |  |  |  |  |  | |  |  |  |  | |  | |  |
| % of which are Sulfatases |  | 0.02 | 0.02 | 0.02 | 0.02 |  | 0.03 |  | 0.08 | | 0.04 | | 0.07 | |  | 0.03 | 0.03 | 0.03 |  | 0.03 | 0.04 | | 0.03 |  | 0.04 | 0.03 | | 0.04 | |  |
|  |  |  |  |  |  |  |  |  |  | |  | |  | |  |  |  |  |  |  |  | |  |  |  |  | |  | |  |
|  |  |  |  |  |  |  |  |  |  | |  | |  | |  |  |  |  |  |  |  | |  |  |  |  | |  | |  |
| *Sequence data represents the 3-0.22 µm size fraction. *Open reading frames (ORFs) of both Eukaryotes and Prokaryotes.* | | | | | | | | | | | | | | | | | | | | | |  |  |  |  |  |  |  |  |  |
